# Hydrogel-Wrapped *Calendula officinalis* L. extracellular vesicles - A novel approach to enhance fracture healing by Macrophage reprogramming

**DOI:** 10.1016/j.mtbio.2025.102592

**Published:** 2025-11-28

**Authors:** Xuesong Wang, Zikun Xie, Riyue Wu, Pengcheng Gao, Liqin Chen, Junlin Zhong, Shuofei Yang, Lei Jin

**Affiliations:** a Department of Stomatology, Jinling Hospital, Affiliated Hospital of Medical School, Nanjing University, Nanjing, 210016, China; b Department of Stomatology, the First Affiliated Hospital of Anhui Medical University, Hefei, 230022, China; c Division of Orthopaedic Surgery, Department of Orthopedics, Nanfang Hospital, Southern Medical University, Guangzhou, Guangdong, China; d Southern Medical University, Guangzhou, Guangdong, 510515, China; e Department of Ultrasound, Third Affiliated Hospital, Sun Yat-Sen University, Guangzhou, China; f Department of Vascular Surgery, Renji Hospital, School of Medicine, Shanghai Jiao Tong University, Pujian Road 160, Shanghai, 200127, China; g Department of Stomatology, The Second Affiliated Hospital Zhejiang University School of Medicine, Hangzhou, 310003, China

**Keywords:** Fracture healing, Single-cell RNA sequencing, Macrophage polarization, *Calendula officinalis* L. -derived extracellular vesicles, Hydrogel

## Abstract

Fracture healing is a complex process often complicated by nonunion and delayed healing, with macrophage polarization being a key regulator of inflammation and tissue repair. Single-cell RNA sequencing (scRNA-seq) has identified genes highly expressed in M1 macrophages at fracture sites, which are closely linked to macrophage polarization. Plant-derived extracellular vesicles (EVs) have shown potential as anti-inflammatory agents but are limited by short duration of action and poor targeting. In this study, we used scRNA-seq to elucidate the mechanisms of M1 macrophage polarization during fracture healing and identified MMP12 as a target regulated by *Calendula officinalis* L.-derived EVs (COEVs) through network pharmacology. To enhance the therapeutic potential of COEVs, we developed a reactive oxygen species (ROS)-responsive hydrogel encapsulating COEVs modified with phosphatidylserine (PS) for macrophage targeting. The hydrogel demonstrated excellent mechanical properties, injectability, self-healing, and ROS responsiveness. Comprehensive in vitro and in vivo experiments confirmed its biocompatibility, ability to regulate target genes, macrophage reprogramming, and osteogenic promotion, offering a novel therapeutic approach for fracture repair.

## Graphical abstract

**Figure:**
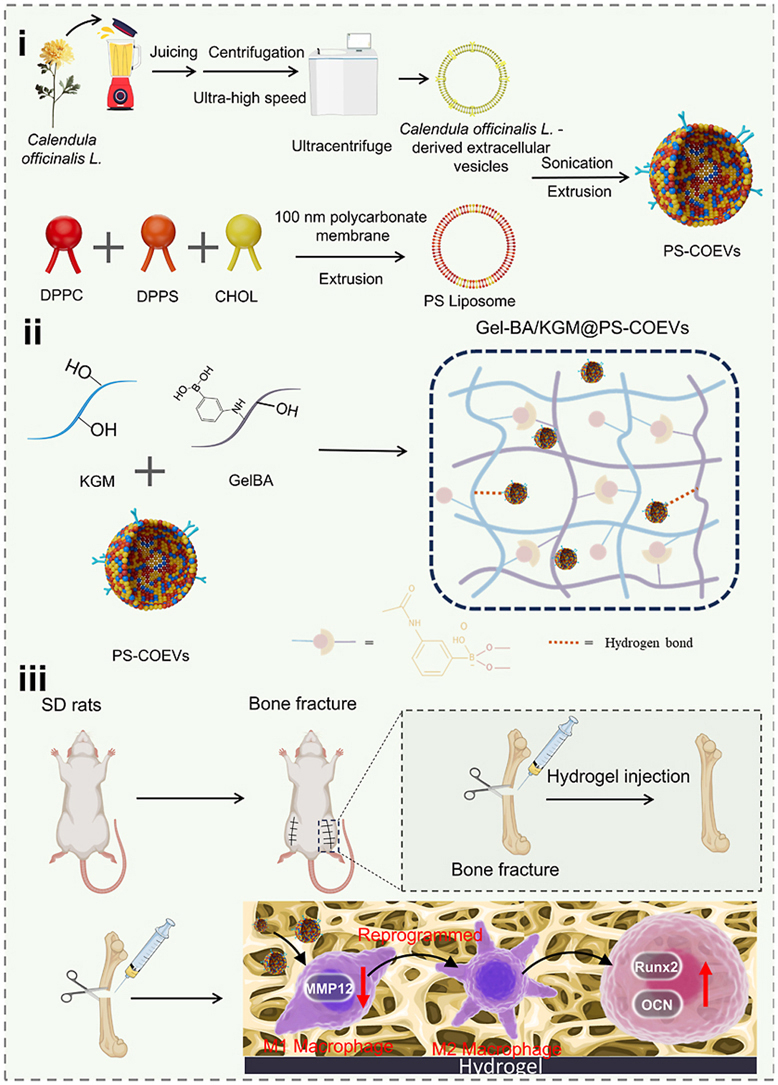

## 1 Introduction

Fracture healing is a complex biological process that is frequently associated with significant risks and challenges, such as nonunion and delayed healing[[1], [2], [3]]. Osteoblasts play a crucial role in fracture healing[[4], [5], [6]], and numerous studies have demonstrated that the inflammatory immune microenvironment at fracture sites affects osteoblast function during this process [7,8]. Macrophages, as key immune cells, are involved in the regulation of inflammation and tissue repair during fracture healing[[9], [10], [11], [12]]. The polarization of macrophages, particularly the shift from the pro-inflammatory M1 phenotype to the anti-inflammatory M2 phenotype, has been shown to have a promising impact on fracture healing[[13], [14], [15]]. However, the precise mechanisms underlying macrophage polarization and its regulation in the context of fractures remain to be fully elucidated.

Advancements in biotechnology, particularly the emergence of single-cell sequencing, have revolutionized our understanding of disease pathogenesis by providing a more detailed and comprehensive view of cellular heterogeneity and gene expression profiles[[16], [17], [18]]. Single-cell RNA sequencing (scRNA-seq) has become an indispensable tool in various disease research fields, including cancer, neurodegenerative diseases, and autoimmune disorders[[19], [20], [21], [22]]. It allows for the identification of rare and novel cell types and states, and provides insights into the molecular mechanisms underlying disease progression and therapeutic responses. To investigate the changes in macrophages during fracture healing, single-cell sequencing technology was employed to identify highly expressed genes in M1-type macrophages within the fracture site. These genes were found to be closely associated with macrophage polarization, shedding light on the potential molecular pathways involved in the regulation of macrophage behavior during fracture healing.

Compared with mammalian or bacterial vesicles, plant-derived extracellular vesicles (EVs) are considered safer and more accessible because they are free of human pathogens, can be produced in large quantities from edible crops, and have built-in anti-inflammatory phyto-cargo[[23], [24], [25], [26], [27]]. Within this plant-EV group, *Calendula officinalis* L. EVs (COEVs) occupy a special niche: our previous network-pharmacology plus single-cell sequencing study specifically matched COEV components to M1-type macrophage-related genes that drive fracture healing [28,29]. While ginger-derived nanoparticles protect against alcohol-induced liver damage [30] and red cabbage-derived EVs modulate gut macrophage polarization [31,32], these reports do not link their cargo to skeletal-relevant gene networks. General reviews further attribute broad anti-inflammatory activity to *Calendula officinalis* L. triterpenoids[[33], [34], [35]], a molecular feature not highlighted for other plant EVs [[36], [37], [38]]. Therefore, the existing literature positions COEVs as the plant-EV candidate with the clearest gene-level hypothesis for targeting M1 macrophages during bone repair. However, plant EVs face several limitations, such as a short duration of action and lack of cell targeting, which hinder their therapeutic efficacy [39].

Current clinical translation of plant extracellular vesicles (EVs) is hindered by two persistent gaps: (i) their half-life in vivo is short because unprotected EVs are rapidly cleared from the fracture site [39], and (ii) they lack intrinsic affinity for the M1-macrophage-rich inflammatory niche [28,29]. Hydrogels have garnered substantial attention as a versatile class of tissue-engineering materials precisely because their remarkable capacity for drug loading and sustained release can bridge these pharmacokinetic and targeting limitations [[40], [41], [42], [43]]. We therefore designed a ROS-responsive hydrogel that exploits the high-ROS inflammatory microenvironment typical of fracture sites[[44], [45], [46], [47]]. To achieve on-demand release of encapsulated plant EVs. To further couple release with cell-specific uptake, the EVs were pre-coated with phosphatididylserine (PS), a ligand that confers macrophage-targeting capability [48,49]. By integrating local, ROS-triggered delivery with PS-mediated macrophage homing, the system directly addresses the dual challenges of short duration and poor targeting that currently limit plant EV efficacy in fracture repair.

In this study, we first elucidated the mechanisms underlying M1 macrophage polarization in the inflammatory immune microenvironment during fracture healing using single-cell sequencing. Subsequently, through the integration of network pharmacology, we identified MMP12 as a target in COEVs that can regulate macrophage polarization during fracture healing. This was further validated through in vitro experiments, which confirmed the regulatory effects of COEVs on the target and their reprogramming role in macrophage polarization. To address the limitations of short duration of action and poor targeting of plant EVs, we designed a ROS-responsive hydrogel encapsulating COEVs modified with PS. The comprehensive evaluation of this hydrogel included its microstructure, mechanical properties, injectability, self-healing, swelling and adhesion properties, as well as its ROS responsiveness and the release behavior of plant EVs. Both in vitro and in vivo experiments demonstrated the hydrogel's excellent biocompatibility, ability to regulate target genes, reprogramming of macrophages, and osteogenic promotion.

## 2 Results and discussion

### 2.1 Identification of Infiltrating cell types in nonunion fractures

To investigate cellular composition changes in nonunion fractures, we integrated transcriptional data from all collected cells. UMAP clustering was applied, generating a two-dimensional projection (Fig. 2A and B). Cell types were annotated by comparing cluster-specific marker genes (Fig. S1). We identified 10 known cell types and one unclassified cell type, including: Osteoblasts (COL1A1, COL1A2), T cells (CD8A, CD8B), Macrophages (CD68), Neutrophils (AZU1, MPO), Erythrocytes (HBB, HBD), Endothelial cells (PECAM1), Smooth muscle cells (MYH11, CNN1), B cells (MS4A1), Plasma cells (MZB1, XBP1), Proliferating cells (MKI67, CDK1) (Figs. S1 and 2). Compared to the control group, macrophages and osteoblasts were more prevalent in the nonunion group (Fig. 2C and D). This finding is consistent with previous studies that have shown an increased presence of macrophages and osteoblasts in nonunion fractures[[9], [10], [11], [12]], suggesting that these cells may play a crucial role in the pathogenesis of nonunion. By comparing our data with previous studies on macrophage polarization, we classified three macrophage subtypes: classically activated M1 macrophages, alternatively activated M2 macrophages, and unpolarized M0 macrophages (Fig. 2E). Differential expression analysis identified significantly altered genes in M1 macrophages (Fig. 2F). Using the CellChat package, we analyzed cell-cell communication networks within the scRNA-seq data. The interaction frequency among four major cell types in normal and nonunion samples is shown in Fig. 2G. Further comparison of interaction strength and frequency revealed an increased osteoblast-M1 macrophage interaction in nonunion fractures (Fig. 2H). This increased interaction frequency suggests that M1 macrophages may be actively influencing osteoblast function in nonunion fractures. Moreover, osteoblasts in nonunion tissue received more incoming signaling compared to controls (Fig. 2I), indicating that M1 macrophages may be contributing to the impaired bone healing process through their interactions with osteoblasts. Gene Ontology Biological Process (GO-BP) and Kyoto Encyclopedia of Genes and Genomes (KEGG) enrichment analyses were conducted on differentially expressed genes (DEGs) in M1 macrophages. Both analyses revealed that the top 15 enriched pathways were predominantly involved in immune and inflammatory responses, with inflammation-related pathways highlighted (Fig. 2J and K). To further investigate pathway-level changes, gene set enrichment analysis (GSEA) was performed to compare M1 macrophages from the nonunion fracture group and the normal-healing group. The results showed that several key inflammatory pathways, including the TNF signaling pathway, NF-κB signaling pathway, and IL-17 signaling pathway, were significantly enriched in the nonunion group (Fig. 2L–N). These findings suggest that M1 macrophages in nonunion fractures exhibit heightened pro-inflammatory signaling, which may contribute to persistent inflammation and impaired fracture healing.

### 2.2 Characterization of PS-COEVs

The preparation of PS-COEVs is shown in Fig. 1. Metabolomic analysis revealed that COEVs were enriched in lipids and lipid-like molecules (28.4 %), Organic acids and derivatives (22.8 %), Triterpenes (16.6 %), and Flavonoid (8.6 %) (Fig. S3). These metabolites are involved in cellular biofunctions such as antioxidation, energy generation, and tissue protection, which may play pivotal roles in fracture healing. GO enrichment analysis of upregulated genes in M1 macrophages revealed that the top 20 enriched pathways were primarily associated with inflammatory response regulation and oxidative stress modulation (Fig. S4). It indicates that COEVs have the potential to regulate the function of M1 macrophages. To identify potential therapeutic targets of COEVs in M1 macrophages, we analyzed 10 active compounds using SwissTargetPrediction and CTD, identifying 587 potential targets (Fig. S5). This suggests that the identified genes may be involved in the pro-inflammatory actions of M1 macrophages and could serve as potential targets for therapeutic interventions. Overlapping these targets with upregulated genes in M1 macrophages, we identified 32 common genes (Fig. 3A). Fig. 3B highlights the top 10 differentially expressed intersecting genes. Through in vitro Real-time quantitative PCR (RT-qPCR) experiments (Mean of control: 1.000, Mean of M1: 1.688, 95 % CI: 0.5814 to 0.7946) (Fig. 3C), immunofluorescence (IF) experiments (Mean of control: 5.307, Mean of M1: 9.500, 95 % CI: 2.472 to 5.915) (Fig. 3D and Fig. S6) and western blot (WB) experiments (Mean of control: 114.3, Mean of M1: 204.0, 95 % CI: 52.72 to 126.6) (Fig. S7), it was confirmed that the expression of MMP12 was most significantly elevated in M1 macrophages, indicating that MMP12 is likely a target for COEVs to regulate the function of M1 macrophages. Zeta potential and particle size distribution of PS, COEVs, and PS-COEVs were measured using a Zetasizer, as shown in Fig. 3E and F. Both PS and COEVs exhibited negative zeta potential, with the zeta potential of the mixed PS-COEVs falling between that of PS and COEVs. The particle size distribution revealed that the average particle sizes of PS and COEVs were 122.0 nm and 92.9 nm, respectively, whereas the average particle size of PS-COEVs was 139.4 nm, larger than both PS and COEVs. Furthermore, the microscopic morphology of PS-COEVs was analyzed using SEM. As illustrated in Fig. 3G, PS-COEVs exhibited a typical circular morphology with a particle size of approximately 150 nm. To verify the better phagocytic ability of PS-COEVs by macrophages, as shown in Fig. 3H, the red signal was stronger in the macrophages of the PS-COEVs group. Consistent with prior reports that PS-bearing vesicles are preferentially engulfed by macrophages [50,51], our findings corroborate the superior phagocytic uptake of PS-decorated carriers. In addition, PS COEVs have a stronger ability to regulate M1 macrophage reprogramming, as shown in Fig. 3I. The marker inducible nitric oxide synthase (iNOS) of M1 macrophages had the weakest signal in the PS-COEVs treatment group.

**Fig. 1:**
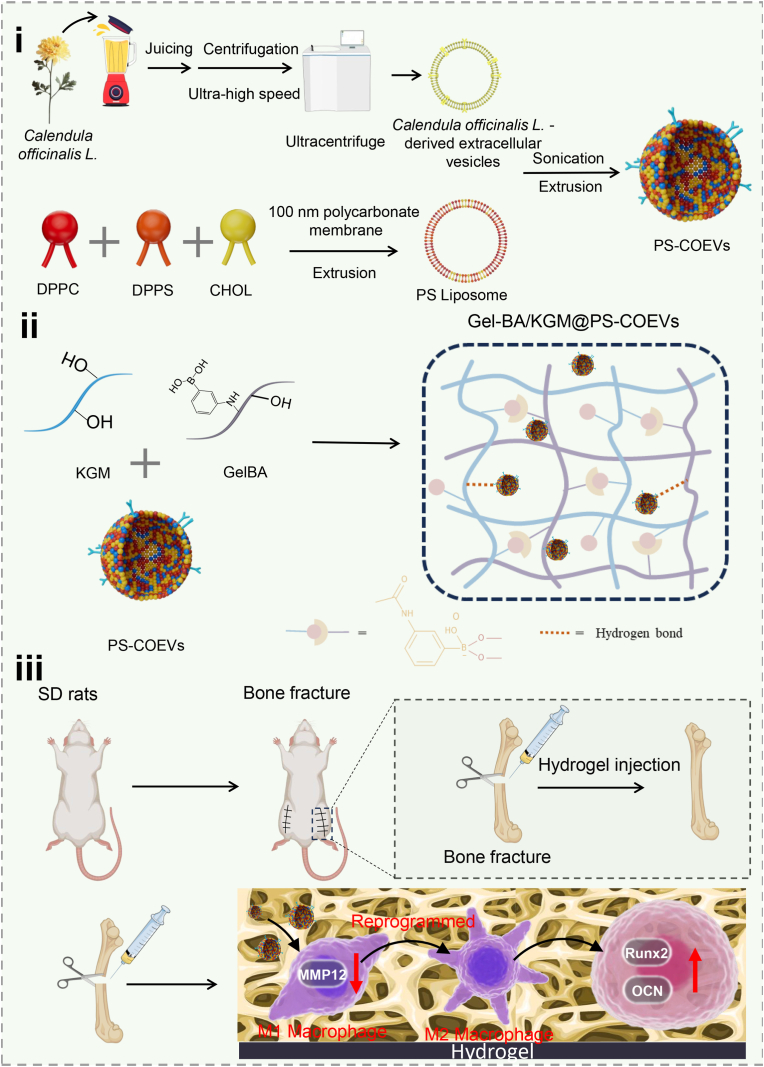
Gel-BA/KGM@PS-COEVs with the ability to promote bone regeneration.

**Fig. 2:**
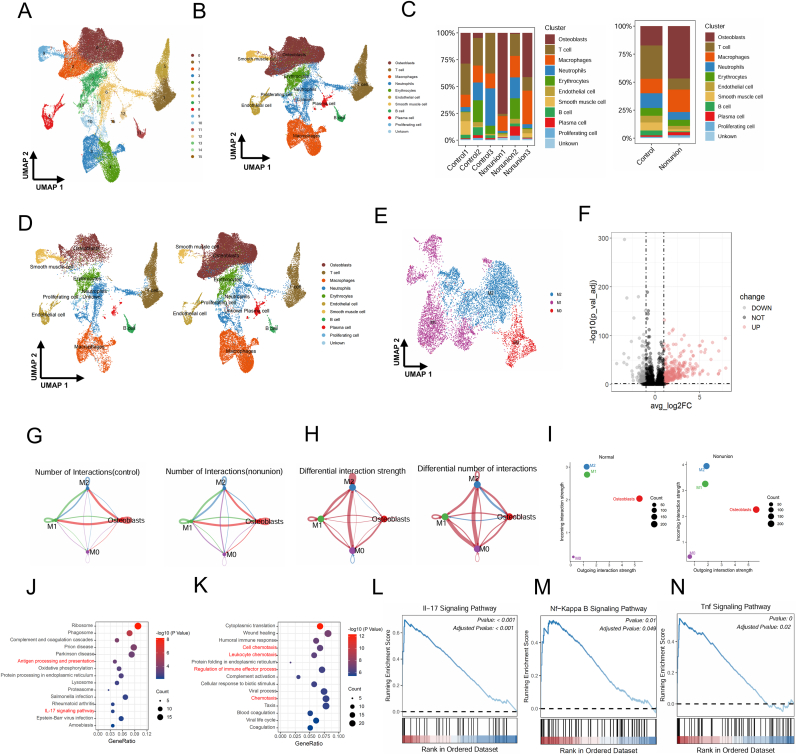
Comprehensive summary of the single-cell atlas from nonunion fracture and normal samples. (A) UMAP plot visualizing the classification of single-cell data into 16 distinct clusters. (B) Identification of 10 different cell types based on marker gene expression analysis. (C) Proportional distribution of various cell types in the nonunion and normal groups. (D) UMAP clustering analysis comparing single-cell profiles of normal and nonunion tissues. (E) UMAP plot displaying three macrophage subpopulations. (F) Volcano plot of differentially expressed mRNAs in M1 macrophages (p < 0.05). (G) Interaction network count plot comparing normal and nonunion groups, where thicker lines represent a higher frequency of interactions between cell types. "Count" denotes the number of interactions. (H) Cell-cell interaction network comparison between the nonunion and normal groups: red lines indicate stronger interactions in the nonunion group, blue lines indicate stronger interactions in the control group, and thicker lines denote greater changes in interaction intensity. (I) Scatter plot illustrating variations in incoming and outgoing signaling among different cell types in the normal and nonunion groups. (J) GO-BP enrichment analysis of DEGs in M1 macrophages. The top 15 significantly enriched pathways are shown. Inflammatory-related pathways are highlighted in red. (K) KEGG enrichment analysis of DEGs in M1 macrophages. The top 15 significantly enriched pathways are shown. Inflammatory-related pathways are highlighted in red. (L–N) GSEA analysis of M1 macrophages comparing the nonunion fracture group with the normal-healing group, showing normalized enrichment scores (NES) of 2.19 (adjusted p < 0.001) in panel L, 1.77 (adjusted p = 0.049) in panel M, and 1.88 (adjusted p = 0.02) in panel N.

**Fig. 3:**
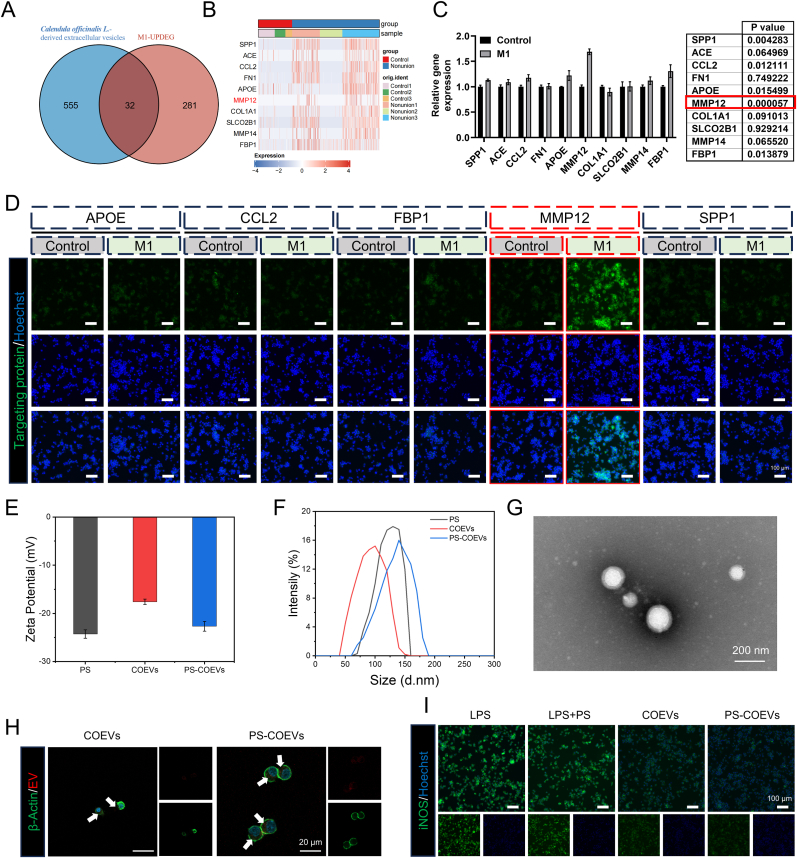
Analysis of active compounds and target genes. (A) Venn diagram illustrating the overlap between drug target genes and upregulated genes in M1 macrophages. (B) Heatmap showing the top 10 intersecting genes with the most significant differential expression. (C) RT-qPCR was utilized to assess the mRNA expression levels of the top 10 differentially expressed genes. (D) IF was utilized to assess the protein expression levels of the differentially expressed genes. (E) Zeta potential of PS, COEVs, and PS-COEVs. (F) Particle size of PS, COEVs, and PS-COEVs. (G) SEM image of PS-COEVs. (H) IF images of Raw264.7 cells incubated with different formations for 1 h, in which COEVs and PS-COEVs were labeled with rhodamine b (red), Raw264.7 cells were labeled with β-Actin (green), and cell nuclei were labeled with Hoechst (blue). (Scale bar: 20 μm) (I) IF was employed to visualize the expression of iNOS in RAW264.7 cells cultured under various conditions. (Scale bar: 100 μm).

### 2.3 Synthesis and characterization of hydrogel

The synthesis of Gel-BA is illustrated in Fig. 4A, where 3-carboxy-phenylboronic acid is activated using EDC and NHS, and BA groups are subsequently grafted into the Gel skeleton via an amide reaction. The incorporation of functional groups was confirmed by Fourier transform infrared (FTIR) spectroscopy and nuclear magnetic resonance (NMR) spectroscopy. As shown in Fig. 4B, the absorption peak of the carbonyl group (C=O) is observed at 1671 cm^−1^, and the characteristic vibration peak corresponding to the bending of C=C bonds in the benzene ring is observed at 1550 cm^−1^. Additionally, stretching vibrations associated with the B-O bond in boric acid were detected at 1349 cm^−1^. The peaks of 7.3–8.5 ppm in the ^1^H NMR spectrum of Gel-BA correspond to the four aromatic protons on the aromatic ring of 3-carboxy-BA (Fig. 4C), thereby confirming the successful conjugation of 3-carboxy-BA. The methyl proton peak of the valine residue side chain in gelatin at 0.9 ppm was chosen as the reference peak to calculate the grafting degree of 3-carboxy-BA, which was determined to be 31.57 %. The chemical shift range of 7.3–8.5 ppm in the ^1^H NMR spectrum of Gel-BA indicates (Fig. 4C) the presence of phenyl protons, confirming successful coupling. The Gel-BA/KGM hydrogel was formed by mixing the Gel-BA and KGM solutions and adjusting the pH to 7.5–8.5 using NaOH, as shown in Fig. 4D. To verify the mechanism of hydrogel formation, FT-IR spectroscopy was conducted to characterize the hydrogels, as shown in Fig. 4E. The B–O stretching vibration of Gel-BA at 1349 cm^−1^ nearly disappears, suggesting that KGM forms a covalent bond with the BA group of Gel-BA. Furthermore, a new peak appeared at 1079 cm^−1^, which corresponds to the B–O–C stretching vibration of the borate ester bond, confirming that the BA group of Gel-BA forms a reversible borate bond with the cis-diol groups of KGM. By investigating the effects of hydrogels encapsulating different concentrations of PS-COEVs on the viability of bone marrow mesenchymal stem cells (BMSCs) (Fig. S8), we found that concentrations exceeding 40 μg/mL of PS-COEVs exhibited an inhibitory effect on BMSC viability. Consequently, 40 μg/mL was determined to be the optimal concentration of PS-COEVs encapsulated within the final hydrogel formulation. The microstructure of the hydrogel was characterized by SEM, as shown in Fig. 4F. Both Gel-BA/KGM and Gel-BA/KGM@PS-COEVs hydrogels exhibited three-dimensional network structures, and the addition of PS-COEVs had almost no effect on the network of the hydrogels. The presence of PS-COEVs can be clearly observed in the SEM magnification image of Gel-BA/KGM@PS-COEVs. In addition, 3D immunofluorescence images (Fig. 4G) showed that PKH26-labeled PS-COEVs were uniformly distributed in the hydrogel.

**Fig. 4:**
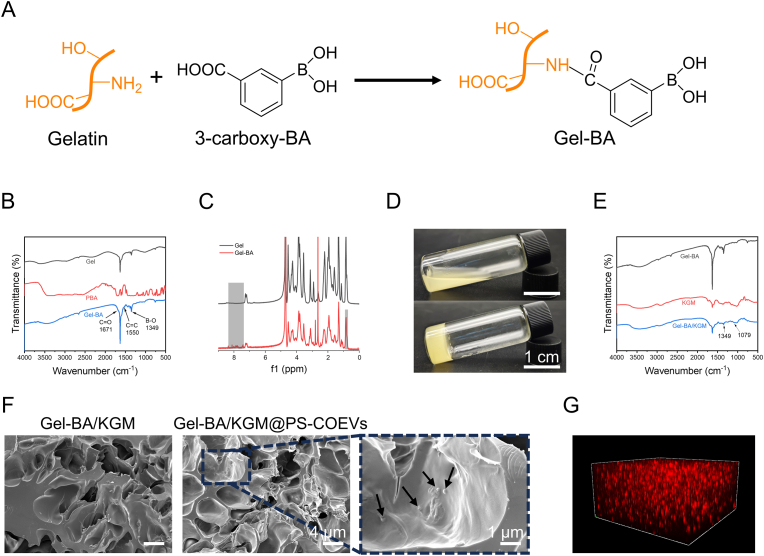
Preparation and characterisation of hydrogels. (A) Mechanism of Gel-BA synthesis. (B) FT-IR spectra of Gel, PBA, and Gel-BA. (C) ^1^H NMR spectra of Gel-Ba and Gel. (D) Preparation of Gel-BA/KGM. (E) FT-IR spectra of Gel-BA, KGM, and Gel-BA/KGM hydrogels. (F) SEM image of the hydrogels. (G) 3D immunofluorescence image of PS-COEVs in hydrogel.

### 2.4 Rheological analysis of hydrogel

The boric acid group of Gel-BA and the cis-diol group of KGM form dynamic borate ester bonds and hydrogen bonds, which endow the hydrogels with stable mechanical properties. The modulus and mechanical properties of the hydrogel were evaluated by rheological analysis, as illustrated in Fig. 5A. In the frequency scanning test, the storage modulus (G′) exceeded the loss modulus (G″) at all frequencies, indicating that the hydrogel behaved as a stable elastic solid. Moreover, the addition of PS-COEVs did not significantly alter the modulus of the hydrogel. During femoral defect repair, the hydrogel requires sufficient elasticity to resist potential deformation. As shown in Fig. 5B and C, the critical strain points of Gel-BA/KGM and Gel-BA/KGM@ PS-COEVs hydrogels were 240 % and 191 %, respectively. Before reaching these critical strain points, the storage modulus (G′) of the hydrogels was greater than the loss modulus (G″), demonstrating the hydrogels’ capacity to withstand strain to a certain extent.

**Fig. 5:**
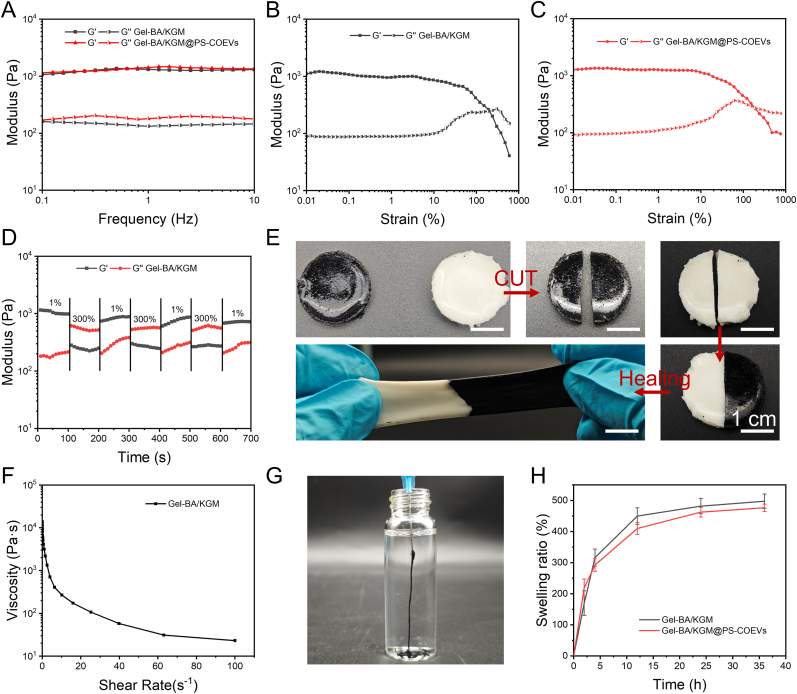
Rheological analysis and adhesion characterisation of hydrogels. (A) Frequency scan testing of Gel-BA/KGM and Gel-BA@PS-COEVs hydrogels. (B) Strain scanning tests of Gel-BA/KGM hydrogel. (C) Strain scanning tests of Gel-BA/KGM@PS-COEVs hydrogel. (D) Alternating strain amplitude scan test of Gel-BA/KGM hydrogel. (E) Images of the self-healing behavior of the hydrogel. (F) Shear thinning test of Gel-BA/KGM hydrogel. (G) Image of the hydrogel extruded from the syringe. (H) Swelling behavior of hydrogels.

The abundant dynamic borate bonds and hydrogen bonds conferred excellent self-healing properties to the hydrogel [52]. This was further confirmed by the alternating strain amplitude scan test (Fig. 5D). At a small strain (1 %), the hydrogel maintained a stable modulus (G' > G″), but when the strain reached 300 %, the dynamic bonds broke, and the network structure collapsed. Upon restoring the strain to 1 %, the dynamic bonds rapidly re-cross-linked, allowing self-healing to occur (G' > G″). Fig. 5E visually demonstrates this phenomenon: two pieces of hydrogel, dyed black and white, were cut in half and reassembled. The dynamic borate bonds enabled the reassembled hydrogel to heal seamlessly and withstand stretching without breaking, further demonstrating its remarkable self-healing performance.

Additionally, the presence of dynamic bonds conferred excellent injectability to the hydrogel, as shown in Fig. 5F. In the shear thinning experiment, the viscosity of the hydrogel decreased with increasing shear rate [53], confirming its excellent injectability and its ability to be extruded through a syringe (Fig. 5G). The swelling behavior of the hydrogels was tested by the immersion method. As depicted in Fig. 5H, Gel-BA/KGM and Gel-BA/KGM@PS-COEVs hydrogels reached swelling equilibrium after 36 h, with swelling rates of 498 % and 477 %, respectively.

### 2.5 Adhesion characterisation and Responsive Degradability of hydrogel

In practice, hydrogels require excellent adhesive properties to ensure continuous wound treatment. The abundant hydroxyl and boric acid groups confer potential adhesive capabilities to the hydrogel. To evaluate these properties, a bonding shear experiment was conducted using bovine bone slices, as illustrated in Fig. 6A–C. Both Gel-BA/KGM and Gel-BA/KGM@PS-COEVs hydrogels exhibited strong adhesion to bovine bone slices, achieving an adhesion strength of 29.57 kPa (95 % CI: 2.400 to 4.840 kPa). Additionally, an end-to-end lap shear test was performed on bovine bone slices to further assess the hydrogel's adhesive performance, achieving an adhesive strength of up to 24.04 kPa (95 % CI: 5.474 to 6.454 kPa), as shown in Fig. 6D–F. To visually demonstrate the adhesive properties, the hydrogel was used to bond bovine bone sheets and rubber together, demonstrating stable adhesion owing to the presence of abundant hydroxyl and boric acid groups (Fig. 6G). Notably, the hydrogel exhibited remarkable adhesive performance on porcine skin, glass, and polytetrafluorylene (PTFE) (Fig. 6H). To verify the hydrogel's adhesive performance in practical applications, a burst pressure test was conducted, as illustrated in Fig. 6I. As shown in Fig. 3J, the hydrogel resisted a burst pressure of approximately 25.25 kPa (95 % CI: 3.491 to 6.798 kPa), attributable to the hydrogel's superior mechanical and adhesive properties.

**Fig. 6:**
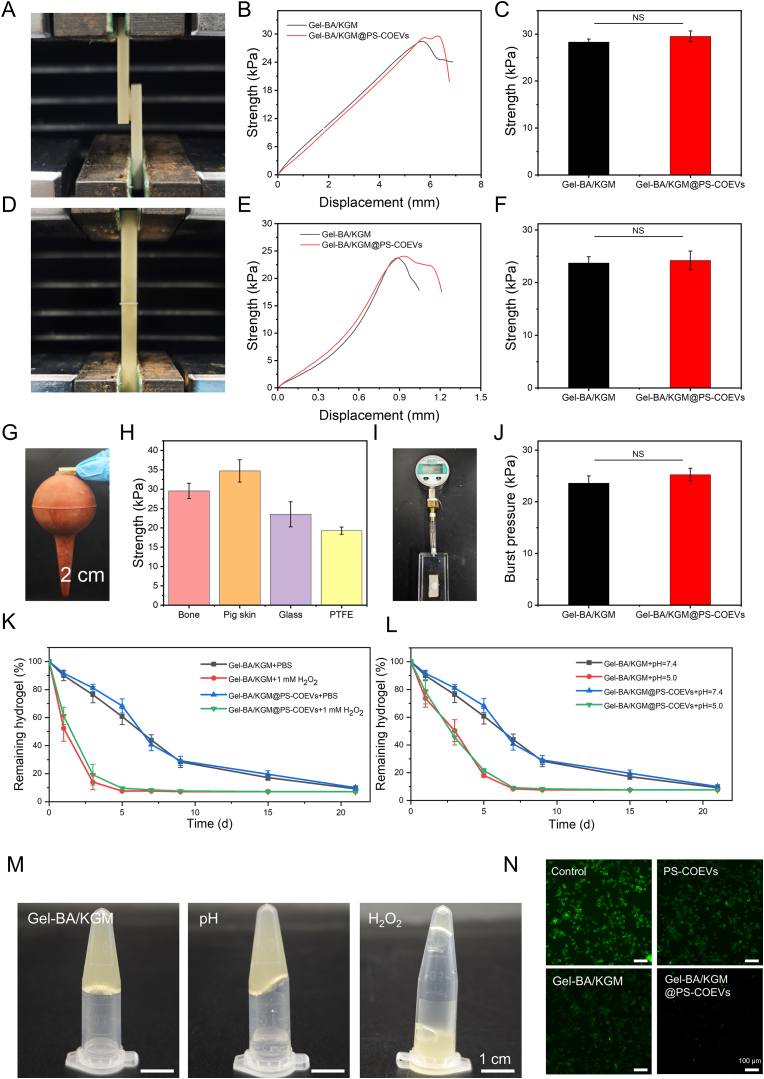
Adhesion Characterizations and Responsive Degradability of Hydrogels. (A) Lap shear test of bovine bone slices. (B) Lap shear curve of bovine bone slices. (C) Adhesion strength of bovine bone slices. (D) end-to-end lap shear test of bovine bone slices. (E) shear curve of end-to-end lap bonding of bovine bone slices. (F) end-to-end adhesion strength of bovine bone slices. (G) Image of the hydrogel adhering bovine bone pieces and rubber together. (H) Adhesion strength of hydrogels to different substrates. (I) Bursting pressure test device for the hydrogel. (J) Bursting pressure of hydrogel. (K) Degradation curves of hydrogels in pH = 7.4 and pH = 5.0 environmental conditions. (L) Degradation curves of hydrogels in an environmental condition with the addition of 100 μL PBS and H_2_O_2_. (M) Representative images of hydrogels treated with 100 μL PBS, HCl (pH = 5.0) and H_2_O_2_ solution. (N) Representative images of intracellular ROS (Scale bar: 100 μm).

The primary cross-linking network of hydrogels is formed by borate ester bonds, which inherently exhibit instability under acidic and ROS conditions, resulting in network collapse and responsive degradation [54]. The responsive degradation behavior of the hydrogel was investigated by treating it with various solutions. As illustrated in Fig. 6K and L, to investigate the degradation performance of the hydrogel under various conditions, we immersed it in different solutions for in vitro degradation experiments and recorded the degradation curves across different pH and ROS levels. The hydrogel exhibited significant degradation in response to both pH and ROS conditions. As shown in Fig. S9A, the alkaline environment (pH = 9.0) inhibited the degradation of hydrogels, whereas the degradation rate increased progressively as the pH decreased from 7.4 to 5.0. Additionally, the degradation rate of the hydrogel could be similarly controlled by adjusting the concentration of H_2_O_2_ (Fig. S9B), with higher concentrations of H_2_O_2_ resulting in a faster degradation rate. Additionally, as shown in Fig. 6M, substantial degradation was observed following treatment with 100 μL HCl (pH = 5.0) or H_2_O_2_ (1 mM), with the H_2_O_2_-treated hydrogel almost completely degraded. This phenomenon occurs due to the irreversible and rapid cleavage of borate ester bonds by H_2_O_2_, leading to accelerated hydrogel degradation in a high ROS environment. The cumulative release profiles of PS-COEVs under different pH and ROS conditions were determined using a fluorescent labeling method, as shown in Fig. S10A. The trend in the cumulative release profiles of PS-COEVs was consistent with the in vitro degradation profiles of the hydrogel, confirming that the hydrogel could responsively release PS-COEVs. Additionally, the stability of PS-COEVs in solutions with different pH and ROS conditions was assessed, as shown in Fig. S10B. Acidic and ROS conditions led to the disintegration of PS-COEVs. The results indicated that, unlike the stable average particle size of PS-COEVs under alkaline and neutral conditions, PS-COEVs in acidic and lower ROS levels (0.5 mM H_2_O_2_) disintegrated and aggregated after 72 h. Remarkably, PS-COEVs were able to maintain their stability for up to 24 h even in a high ROS environment. As shown in Fig. 6N, all three treatment groups exhibited varying degrees of ROS scavenging ability, among which Gel BA/KGM@PS-COEVs group has the strongest ROS scavenging ability. This further confirmed the hydrogel's responsive degradation under acidic and reactive oxygen conditions. As the hydrogel network disintegrated, the release of PS-COEVs accelerated. This responsive degradation facilitates the on-demand release of PS-COEVs, thereby enhancing their efficacy.

### 2.6 Biocompatibility of the gel-BA/KGM@PS-COEVs

To evaluate the biocompatibility of Gel-BA/KGM@PS-COEVs, the viability of bone marrow mesenchymal stem cells (BMSCs) and M1 macrophages were assessed using live/dead staining. Cells were seeded in 24-well and 96-well plates and co-cultured under various conditions, including control, PS-COEVs, Gel-BA/KGM, and Gel-BA/KGM@PS-COEVs. After 24 h of incubation, the distribution of viable cells, marked by green fluorescence, was found to be homogeneously distributed across the entire field of view in both experimental and control groups (Fig. 7A and B). Quantitative analysis of cell viability (Fig. S11 and S12) revealed no significant differences between the Gel-BA/KGM@PS-COEVs group and the control group, indicating that the treatment did not negatively impact cell survival. Additionally, cell adhesion and proliferation were comparable among all groups, with no significant variations observed (Fig. 7C). These results collectively demonstrate the good biocompatibility of the tested implants.

**Fig. 7:**
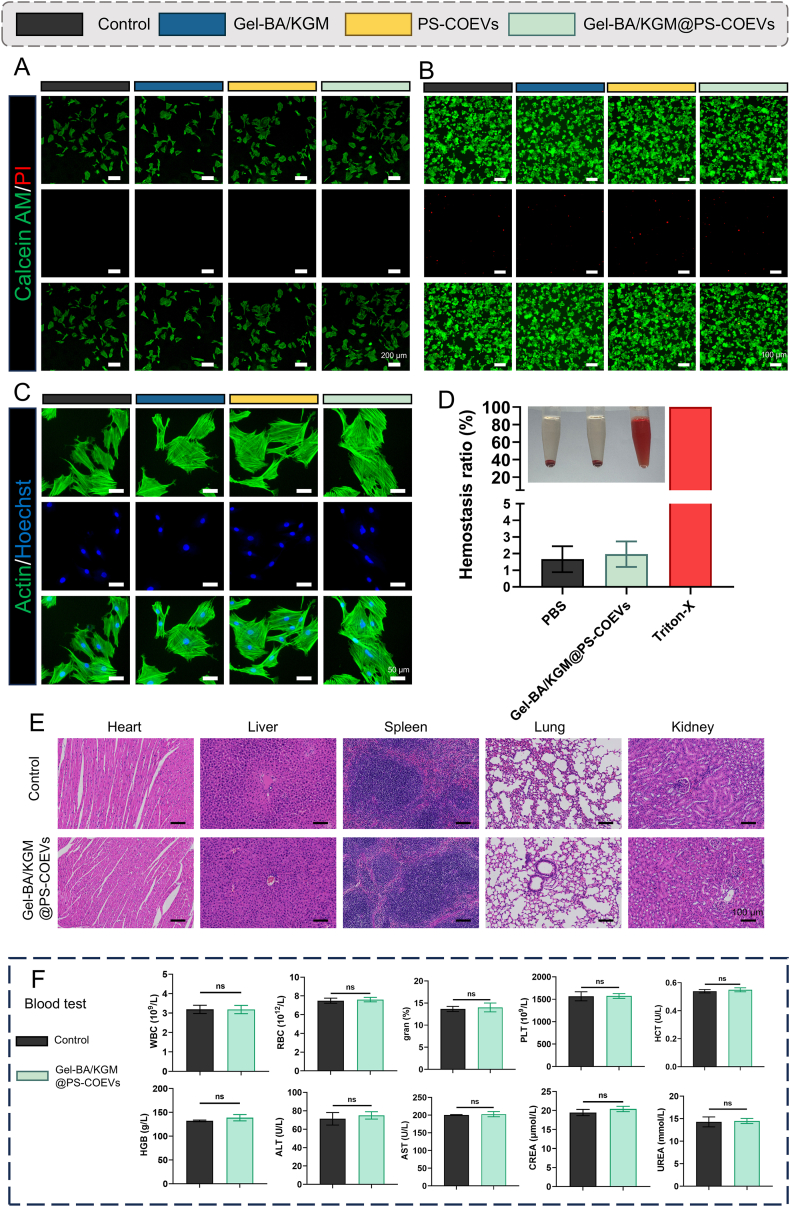
Biocompatibility Evaluation of Gel-BA/KGM@COEVs. (A) Live/Dead staining was conducted across all experimental groups in BMSCs. Viable cells were identified by green fluorescence (calcein AM staining), while non-viable cells were marked by red fluorescence (propidium iodide staining) (Scale bar: 200 μm). (B) Live/Dead staining was conducted across all experimental groups in M1 macrophages (Scale bar: 100 μm). (C) Cytoskeletal morphology of BMSCs cultured under different conditions after 72 h, with a scale bar of 50 μm. (D) Hemocompatibility assessment of Gel-BA/KGM@COEVs, including visual documentation and hemolysis rate quantification (n = 3). (E) Histopathological analysis of major organs from control and Gel-BA/KGM@COEVs-treated rat. (F) Measurement of serum biomarker levels in control and Gel-BA/KGM@COEVs groups. Data are presented as mean ± SD. ns = not significant.

Hemocompatibility of Gel-BA/KGM@PS-COEVs was further assessed via a hemolysis assay, with PBS serving as a negative control and Triton X-100 as a positive control. The solution from the Triton X-100 group exhibited a distinct red color, indicative of hemolysis and erythrocyte lysis, whereas the Gel-BA/KGM@PS-COEVs group maintained a clear appearance similar to the negative control (95 % CI: 12.60 to 11.99 %) (Fig. 7D). Quantitative hemolysis data showed that both the Gel-BA/KGM@PS-COEVs and PBS groups had hemolysis rates below the 5 % threshold, suggesting excellent hemocompatibility. These findings highlight the minimal hemolytic of Gel-BA/KGM@PS-COEVs, comparable to the PBS control.

BA/KGM@PS-COEVs treatment does not adversely affect renal or hepatic function. Histopathological evaluations were also performed on multiple organ tissues from treated rat, including the liver, kidney, heart, spleen, and lung. First, to evaluate the in vivo distribution of Gel BA/KGM@COEVs, we performed subcutaneous injections in animals and monitored the localization of PS-COOEVs in various vital organs at distinct time points. Our results revealed that PS-COOEVs were predominantly accumulated in the liver, spleen, lungs, and kidneys (Fig. S13). Histological sections (Fig. 7E) revealed no significant pathological alterations or signs of organ toxicity attributable to Gel-BA/KGM@PS-COEVs treatment. The lack of inflammatory or degenerative changes in the examined organs further supports the safety profile of Gel-BA/KGM@PS-COEVs.

To further investigate the in vivo biocompatibility of Gel-BA/KGM@PS-COEVs, subcutaneous injections were administered to rat, with a control group receiving PBS injections. Blood samples were collected from both groups for comprehensive biochemical analysis. Results (Fig. 7F) indicated that Gel-BA/KGM@PS-COEVs treatment did not induce hematologic toxicity. This conclusion was supported by the analysis of various blood parameters, including white blood cell (WBC) count (Mean of control: 3.190 × 10^9^/L, Mean of Gel-BA/KGM@PS-COEVs: 3.180 × 10^9^/L, 95 % CI: 0.6295 to 0.6095 × 10^9^/L), hemoglobin (HGB) (Mean of control: 132.0 g/L, Mean of Gel-BA/KGM@PS-COEVs: 138.7 g/L, 95 % CI: 4.362 to 17.70 g/L), red blood cell (RBC) count (Mean of control: 7.490 × 10^12^/L, Mean of Gel-BA/KGM@PS-COEVs: 7.607 × 10^12^/L, 95 % CI: 0.4598 to 0.6931 × 10^12^/L), platelet (PLT) count (Mean of control: 1567 × 10^9^/L, Mean of Gel-BA/KGM@PS-COEVs: 1574 × 10^9^/L, 95 % CI: 174.1 to 188.8 × 10^9^/L), hematocrit (HCT) (Mean of control: 0.5392 U/L, Mean of Gel-BA/KGM@PS-COEVs: 0.5493 U/L, 95 % CI: 0.01966 to 0.03992 U/L), and granulocyte percentage (gran %) (Mean of control: 13.67 %, Mean of Gel-BA/KGM@PS-COEVs: 14.00 %, 95 % CI: 1.518 to 2.184 %), which showed no significant differences from the control group. Moreover, biochemical markers of liver and kidney function, such as aspartate aminotransferase (AST) (Mean of control: 200.3 U/L, Mean of Gel-BA/KGM@PS-COEVs: 202.7 U/L, 95 % CI: 9.627 to 14.29 U/L), alanine aminotransferase (ALT) (Mean of control: 71.33 U/L, Mean of Gel-BA/KGM@PS-COEVs: 75.00 U/L, 95 % CI: 8.989 to 16.32 U/L), urea (UREA) (Mean of control: 14.30 mmol/L, Mean of Gel-BA/KGM@PS-COEVs: 14.50 mmol/L, 95 % CI: 1.796 to 2.196 mmol/L), and creatinine (CREA) (Mean of control: 19.47 μmol/L, Mean of Gel-BA/KGM@PS-COEVs: 20.40 μmol/L, 95 % CI: 0.7882 to 2.655 μmol/L), were measured and found to be similar between the two groups.

In summary, the comprehensive in vivo and in vitro assessments of Gel-BA/KGM@PS-COEVs demonstrate its potential as a biocompatible therapeutic agent. The treatment exhibited no cytotoxicity no hematologic toxicity, no adverse effects on liver and kidney function, and no signs of tissue damage in vital organs. These findings collectively highlight the favorable biocompatibility of Gel-BA/KGM@PS-COEVs, supporting its potential as a safe and effective therapeutic candidate.

### 2.7 MMP12/NF-κB axis drives M1→M2 repolarisation by gel-BA/KGM@PS-COEVs

To elucidate how Gel-BA/KGM@PS-COEVs reprogram pro-inflammatory macrophages, we used LPS-stimulated RAW264.7 cells as an in vitro M1-polarized model. RT-qPCR revealed a marked down-regulation of MMP12 transcript after treatment with Gel-BA/KGM@PS-COEVs (Mean of control: 1.000, Mean of Gel-BA/KGM@PS-COEVs: 0.3983, 95 % CI: 0.4749 to 0.7365) (Fig. 8A). Emerging evidence implicates MMP12 as a key rheostat of canonical NF-κB–driven macrophage polarization [55]. Accordingly, we interrogated this signalling axis. Western blotting showed a concerted decline in MMP12 abundance and in the phosphorylated pools of p65 and IκBα (Fig. 8B–E), signifying a marked suppression of canonical NF-κB activity (MMP12: Mean of control: 189.0, Mean of Gel-BA/KGM@PS-COEVs: 107.7, 95 % CI: 49.91 to 112.8; p-p65: Mean of control: 179.0, Mean of Gel-BA/KGM@PS-COEVs: 121.0, 95 % CI: 30.88 to 85.12; p-lkba: Mean of control: 171.7, Mean of Gel-BA/KGM@PS-COEVs: 85.67, 95 % CI: 57.86 to 114.1). To further consolidate the MMP12–NF-κB axis, we performed orthogonal perturbation, lentiviral MMP12 over-expression in RAW264.7 cells blunted the Gel-BA/KGM@PS-COEV-induced decrease in p-p65 and largely abolished the accompanying up-regulation of Arg-1 (Fig. S14). Conversely, pre-activation of NF-κB with a selective agonist restored the M1 phenotype even in the continued presence of COEVs (Fig. S14), confirming that NF-κB suppression is necessary for the observed repolarisation.

**Fig. 8:**
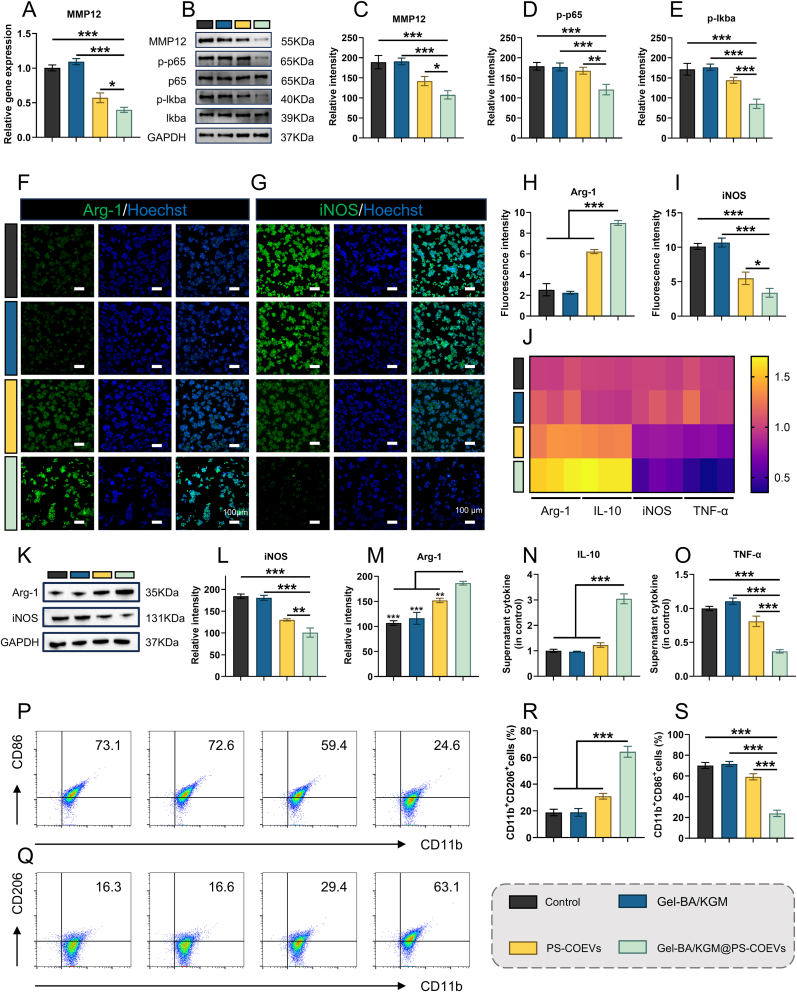
Gel-BA/KGM@PS-COEVs reprogram M1 macrophages via the MMP12/NF-κB axis. (A) Relative MMP12 mRNA levels measured by RT-qPCR (n = 3). (B) Representative Western blot images of MMP12 and NF-κB pathway proteins (p-p65, p-IκBα). (C–E) Quantification of bands shown in (B) (n = 3). (F) Immunofluorescence images of Arg-1 (green) and iNOS (red) in macrophages. (G) Merged images with DAPI counterstain. (H, I) Quantitative fluorescence intensity for Arg-1 and iNOS (n = 3). (J) RT-qPCR heat-map of Arg-1, IL-10, iNOS and TNF-α (n = 3). (K) Western blot of Arg-1 and iNOS. (L, M) Quantification of bands shown in (K) (n = 3). (N, O) ELISA measurement of secreted IL-10 and TNF-α (n = 3). (P) Representative flow cytometry dot-plots of CD11b^+^CD86^+^ and CD11b^+^CD206^+^ macrophages. (Q) Merged flow plots for clarity. (R, S) Percentages of CD11b^+^CD86^+^ and CD11b^+^CD206^+^ cells (n = 3). Statistical significance was determined by One-way ANOVA followed by Bonferroni's multiple comparison test (*P < 0.05, **P < 0.01, and ***P < 0.001).

Functionally, Gel-BA/KGM@PS-COEVs precipitated a robust phenotypic switch. Immunofluorescence showed diminished iNOS-positive and increased Arg-1-positive cells (iNOS: Mean of control: 2.540, Mean of Gel-BA/KGM@PS-COEVs: 8.990, 95 % CI: 7.334 to −5.566; Arg-1: Mean of control: 10.12, Mean of Gel-BA/KGM@PS-COEVs: 3.393, 95 % CI: 4.967 to 8.493) (Fig. 8F–I), mirrored at the transcriptional level by reduced TNF-α/iNOS and elevated IL-10/Arg-1 (Fig. 8J). Western blot and ELISA of conditioned media corroborated the reciprocal cytokine shift (iNOS: Mean of control: 184.3, Mean of Gel-BA/KGM@PS-COEVs: 101.0, 95 % CI: 65.91 to 100.8; Arg-1: Mean of control: 107.0, Mean of Gel-BA/KGM@PS-COEVs: 186.3, 95 % CI: 97.67 to −61.00; IL-10: Mean of control: 1.000, Mean of Gel-BA/KGM@PS-COEVs: 3.045, 95 % CI: 2.338 to −1.752; TNF-α: Mean of control: 1.000, Mean of Gel-BA/KGM@PS-COEVs: 0.3670, 95 % CI: 0.5038 to 0.7622; IL-1β: Mean of control: 1.000, Mean of Gel-BA/KGM@PS-COEVs: 0.5227, 95 % CI: 0.2978 to 0.6569; TGF-β: Mean of control: 1.000, Mean of Gel-BA/KGM@PS-COEVs: 2.251, 95 % CI: 1.586 to −0.9163) (Fig. 8K–O and Fig. S15). Flow-cytometric profiling further evidenced a decline in CD86^+^ M1 cells and a reciprocal rise in CD206^+^ M2 cells (CD86: Mean of control: 70.13 %, Mean of Gel-BA/KGM@PS-COEVs: 23.97 %, 95 % CI: 38.71–53.62 %; CD206: Mean of control: 18.80 %, Mean of Gel-BA/KGM@PS-COEVs: 64.27 %, 95 % CI: 53.25 to −37.68 %) (Fig. 8P–S). These data converge on a mechanistic model wherein Gel-BA/KGM@PS-COEVs silence MMP12, thereby blunting NF-κB signalling and steering M1 macrophages toward an anti-inflammatory M2 phenotype. Besides the MMP12/NF-κB axis validated herein, additional pathways—for example, STAT6 or PI3K/AKT signaling—may also contribute to the COEV-induced M1→M2 shift; dedicated functional studies are required to substantiate these possibilities.

### 2.8 Gel-BA/KGM@PS-COEVs-mediated Macrophage modulation enhances osteogenic behaviors of BMSCs

To further elucidate the impact of Gel-BA/KGM@PS-COEVs-mediated macrophage modulation on the osteogenic behaviors of BMSCs, a cell co-culture system was employed. Macrophages were co-cultured with BMSCs (pre-osteoblasts) using transwell plates to facilitate indirect interaction between the two cell types. Osteogenesis was subsequently assessed via alkaline phosphatase (ALP) activity and Alizarin Red staining (ARS) on days 7 and 14, respectively.

As depicted in Fig. 9A–D, both ALP activity and mineral deposition were significantly elevated in the Gel-BA/KGM@PS-COEVs co-culture group compared to the control groups (ALP: Mean of control: 0.2043 mmol/min, Mean of Gel-BA/KGM@PS-COEVs: 0.3443 mmol/min, 95 % CI: 0.1740 to −0.1060 mmol/min; ARS: Mean of control: 0.5284, Mean of Gel-BA/KGM@PS-COEVs: 1.192, 95 % CI: 0.7674 to −0.5594) and day 0 (Fig. S16). This observation indicates that macrophages, modulated by Gel-BA/KGM@PS-COEVs, enhance in vitro osteogenesis. This finding is demonstrating the positive role of M2 macrophages in promoting osteogenic differentiation.

**Fig. 9:**
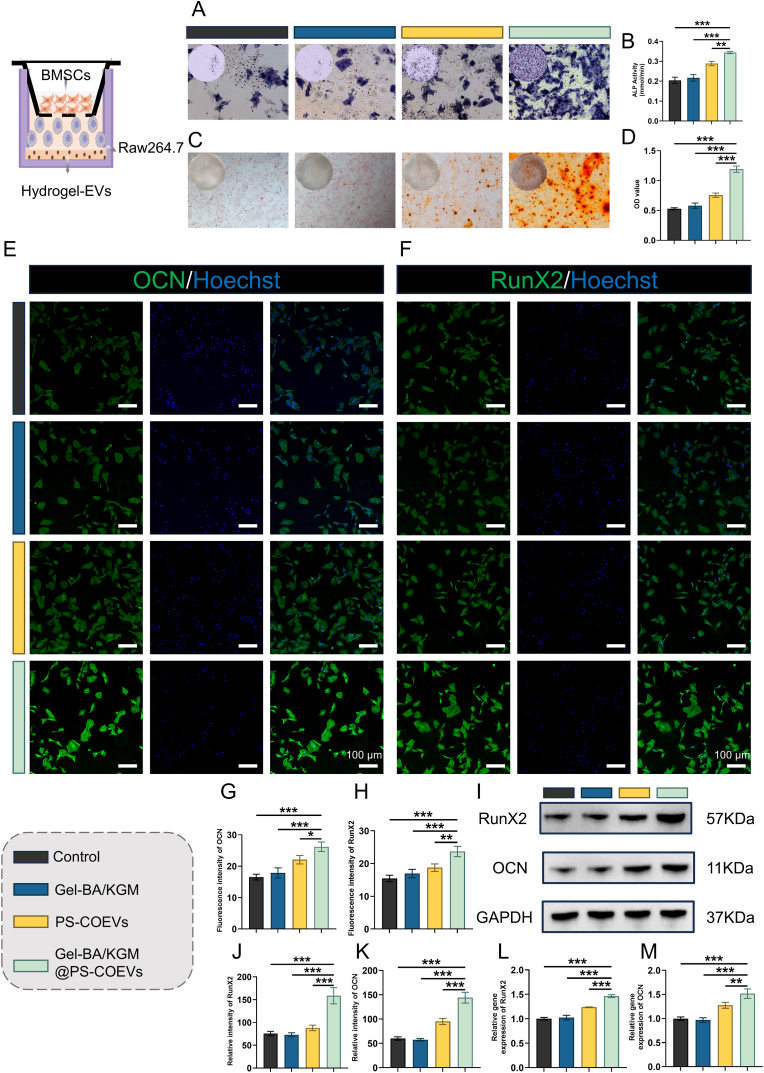
Gel-BA/KGM@PS-COEVs Modulated Osteogenic of BMSCs. (A) ALP activity visualization in BMSCs following 7-day co-culture. (B) Densitometric quantification of ALP activity (n = 3). (C) Calcium deposition patterns revealed by ARS staining after 14-day culture. (D) Spectrophotometric determination of ARS mineralization indices (n = 3). (E, F) Subcellular localization of RunX2 and OCN via immunofluorescence after 7-day induction (Scale bars = 100 μm). (G, H) Fluorescence signal intensity profiling of osteogenic markers (n = 3). (I) WB of RunX2 and OCN protein expression profiles. (J, K) Densitometric evaluation of electrophoretic bands using image analysis software (n = 3). (L, M) Transcriptional level of osteogenesis-associated genes (RunX2, OCN) assessed by RT-qPCR (n = 3). Statistical significance was determined by One-way ANOVA followed by Bonferroni's multiple comparison test (*P < 0.05, **P < 0.01, and ***P < 0.001).

Further molecular analyses were conducted using immunofluorescence, WB, and RT-qPCR to investigate the expression of osteogenesis-related genes. The results revealed that, compared to other groups, the expression levels of key osteogenic markers, such as RunX2 and osteocalcin (OCN), compared with the control group, the Gel-BA/KGM@PS-COEVs group exhibited significant up-regulation of all osteogenic markers. Immunofluorescence revealed that RunX2 rose from 15.47 to 23.66 (95 % CI −11.47 to −4.91) and OCN from 16.54 to 26.20 (95 % CI −13.25 to −6.07). Western blot showed even larger increases: RunX2 from 75.67 to 158.7 (95 % CI −109.0 to −57.0) and OCN from 60.00 to 144.0 (95 % CI −101.6 to −66.4). Likewise, RT-qPCR demonstrated transcript levels of RunX2 increasing to 1.464-fold (95 % CI −0.545 to −0.382) and OCN to 1.516-fold (95 % CI −0.689 to −0.349) relative to the control (Fig. 9E–M). This upregulation of osteogenic genes suggests that Gel-BA/KGM@PS-COEVs-mediated macrophage modulation not only enhances the osteogenic potential of BMSCs but also provides a conducive microenvironment for bone formation.

### 2.9 The in vivo osteogenic and osseointegrative potential of gel-BA/KGM@PS-COEVs

As depicted in Fig. 10A, the surgical procedure for implanting the samples into the femoral bones was meticulously documented. Eight weeks following the surgery, the excised femoral bones were subjected to micro-CT analysis to generate 2D and 3D reconstructions and to quantify the newly formed bone tissue. The vertical section 2D imaging of the implant site revealed that the Gel-BA/KGM@PS-COEVs group exhibited a significantly higher degree of osteogenesis compared to the other three groups and day 0 (Figs. S17 and S18). This trend was corroborated by the 3D reconstructions (Fig. 10B).

**Fig. 10:**
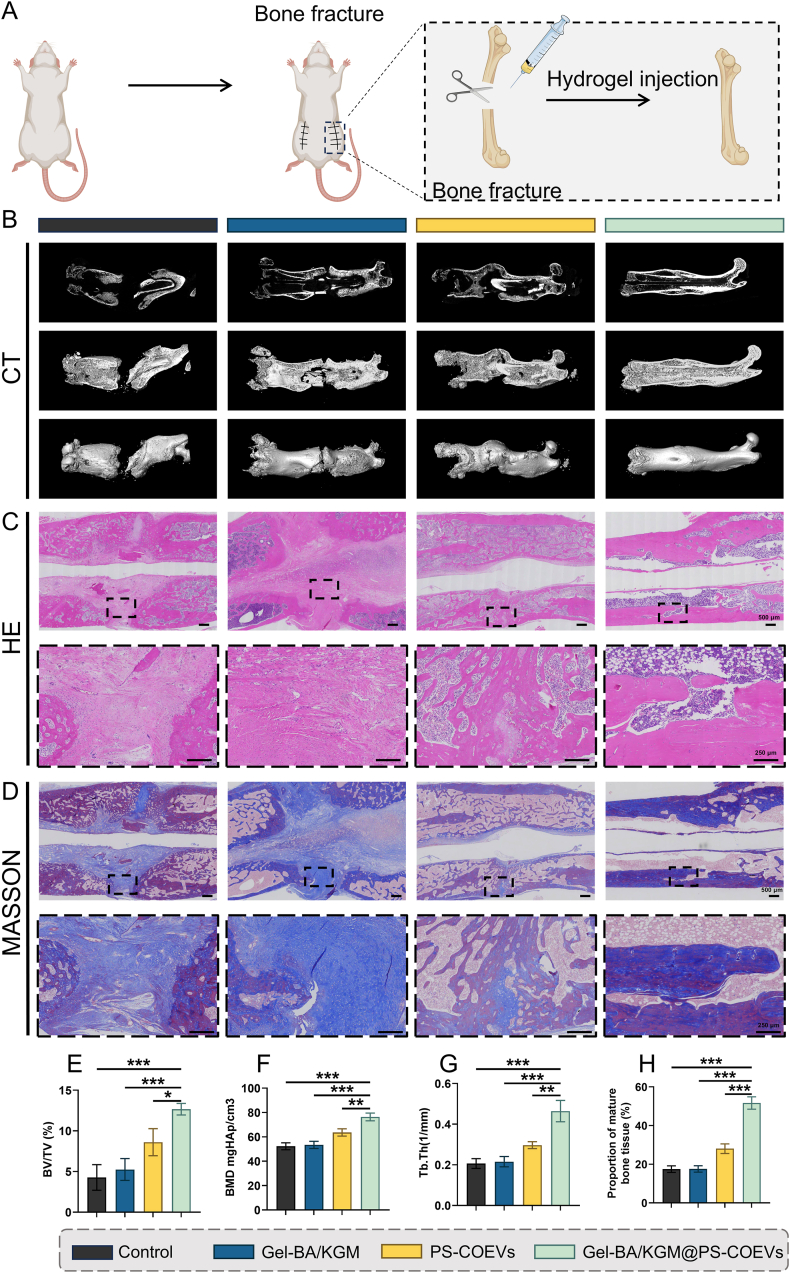
In Vivo Therapeutic Efficacy of Gel-BA/KGM@PS-COEVs Adhesive in rat femoral fracture Model. (A) Surgical workflow schematic: Femoral osteotomy creation and biomaterial implantation for regenerative treatment. (B) Micro-CT reconstruction of osseous regeneration. (C, D) Multi-scale histomorphometric evaluation (H&E: cellular infiltration; Masson's trichrome: collagen organization) at defect site (Scale bars = 250 μm). (E–G) Morphometric quantification of BV/TV, BMD, and Tb.Th (n = 3). (H) Osteogenic maturation index. Statistical significance was determined by One-way ANOVA followed by Bonferroni's multiple comparison test (*P < 0.05, **P < 0.01, and ***P < 0.001).

Quantitative assessments of new bone formation are presented in Fig. 10E–G, encompassing bone volume fraction (BV/TV), bone mineral density (BMD), and trabecular thickness (Tb.Th). The Gel-BA/KGM@PS-COEVs group exhibited the highest values for BV/TV, BMD, and Tb.Th, indicating its superior osteogenic capacity among the tested groups (BV/TV: Mean of control: 4.274 %, Mean of Gel-BA/KGM@PS-COEVs: 12.66 %, 95 % CI: 11.99 to −4.793 %; BMD: Mean of control: 52.30 mgHAp/cm^3^, Mean of Gel-BA/KGM@PS-COEVs: 76.37 mgHAp/cm^3^, 95 % CI: 31.89 to −16.25 mgHAp/cm^3^; Tb.Th: Mean of control: 0.2062 1/mm, Mean of Gel-BA/KGM@PS-COEVs: 0.4642 1/mm, 95 % CI: 0.3433 to −0.1725 1/mm).

To investigate the cellular and molecular mechanisms underlying bone regeneration, decalcified histological sections were subjected to H&E staining, Masson's trichrome staining, and immunohistochemical analysis targeting macrophage polarization markers (iNOS for M1 macrophages, Arg-1 for M2 macrophages) and osteogenic differentiation markers (OCN and RunX2). The H&E staining results corroborated radiographic observations, with the Gel-BA/KGM@PS-COEVs group exhibiting enhanced collagen deposition and increased osteoblast accumulation at the fracture interface compared to controls (Fig. 10C). These histological features were consistent with elevated osteogenic activity and tissue remodeling. Masson's trichrome staining was employed to assess the maturity of new bone formation during the fracture healing process. This staining technique differentiates bone maturity based on collagen organization, with mature bone appearing red and immature bone predominantly blue. The Gel-BA/KGM@PS-COEVs group exhibited the highest collagen deposition, characterized by dense and organized collagen fibers and mature bone deposition, resembling normal bone (Mean of control: 17.47 %, Mean of Gel-BA/KGM@PS-COEVs: 51.69 %, 95 % CI: 40.35 to −28.09 %) (Fig. 10D–H). This suggests that Gel-BA/KGM@PS-COEVs promote the formation of mature bone tissue during the healing process. Immunohistochemical staining for macrophage markers (iNOS for M1 macrophages and Arg-1 for M2 macrophages) was performed to assess the macrophage polarization at the fracture site. The results indicated a shift towards the M2 phenotype in the Gel-BA/KGM@PS-COEVs group, which is associated with anti-inflammatory and tissue repair functions (Arg-1: Mean of control: 14.36 %, Mean of Gel-BA/KGM@PS-COEVs: 70.36 %, 95 % CI: 67.34 to −44.66 %; iNOS: Mean of control: 71.39 %, Mean of Gel-BA/KGM@PS-COEVs: 10.95 %, 95 % CI: 50.90–69.98 %) (Fig. 11A, B, E and F). To visualize the spatial distribution of these polarized macrophages within the callus, we performed dual-label immunofluorescence for F4/80 together with CD86 (M1) or CD206 (M2). Consistent with the IHC data, confocal imaging revealed that F4/80^+^CD86^+^ signals were markedly reduced while F4/80^+^CD206^+^ signals were enriched throughout the endochondral region of the Gel-BA/KGM@PS-COEV-treated defects, confirming that the M2-dominated immune niche overlaps the areas of active osteogenesis (Fig. S19). This polarization is crucial for promoting a conducive microenvironment for bone regeneration. OCN and RunX2 are critical proteins used to stain osteoblasts and osteoids, primarily expressed during bone mineralization. At 8 weeks post-treatment, the expression levels of OCN and RunX2 were significantly higher in the Gel-BA/KGM@PS-COEVs group compared to the other three groups (OCN: Mean of control: 25.03 %, Mean of Gel-BA/KGM@PS-COEVs: 35.08 %, 95 % CI: 15.99 to −4.111 %; RunX2: Mean of control: 10.22 %, Mean of Gel-BA/KGM@PS-COEVs: 15.13 %, 95 % CI: 8.352 to −1.468 %) (Fig. 11C, D, G and H). This elevated expression suggests a superior mineralization ability of Gel-BA/KGM@PS-COEVs in the bone matrix, indicating enhanced osteogenic activity and bone formation. The shift towards an M2 macrophage phenotype in the Gel-BA/KGM@PS-COEVs group suggests that macrophage modulation plays a crucial role in promoting a favorable microenvironment for bone repair (Fig. S20). Collectively, our in-vitro and rodent data position the hydrogel/COEV construct as a candidate for larger pre-clinical studies, not as evidence of clinical efficacy. Remaining hurdles—batch variance, potential immunogenicity and the absence of tailored botanical-EV regulations—underscore the need for standardized isolation protocols and early regulatory engagement, as recently emphasized in The BMJ commentary on linking experimental discovery to population-level health innovation [56]. Extending this bench-to-population vision will require coordinated multi-centre validation and health-economic modelling to ensure equitable, global access to plant-EV fracture therapeutics.

**Fig. 11:**
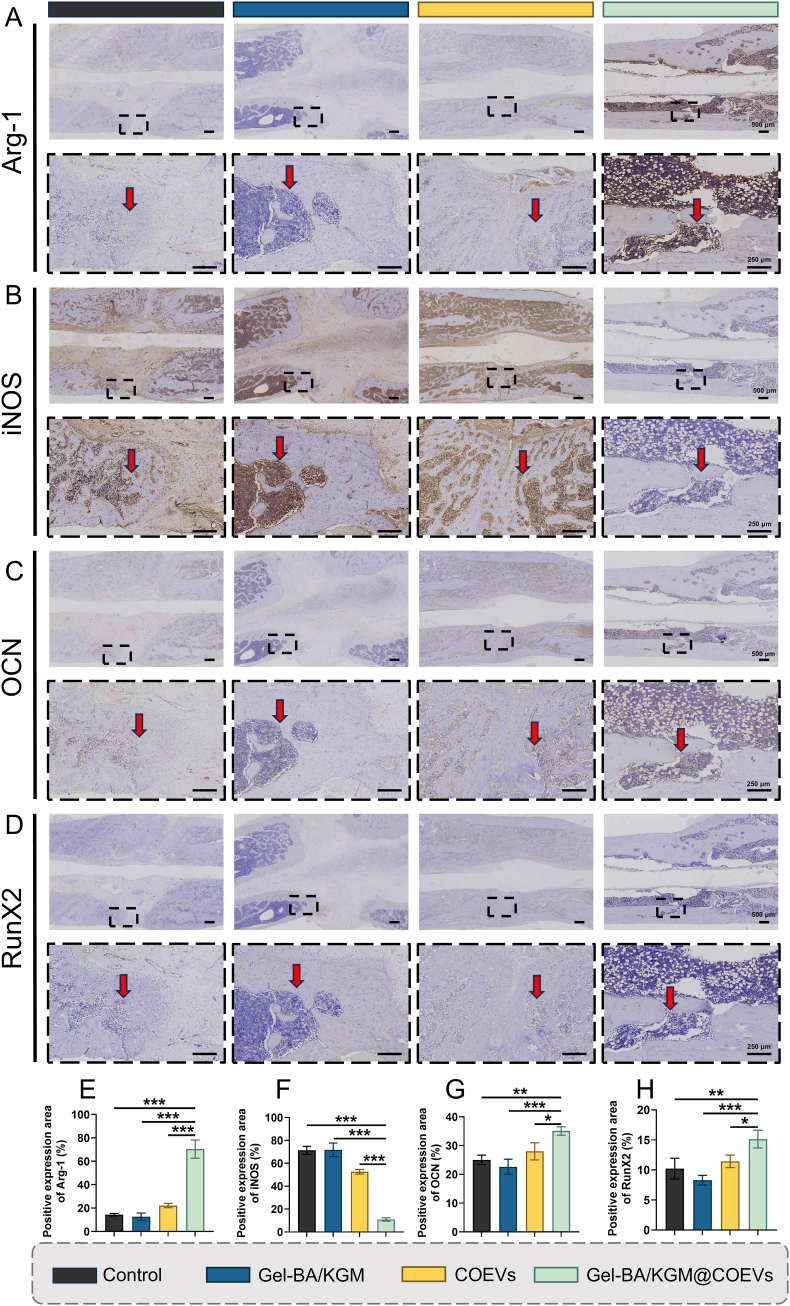
Histological analysis of osteogenesis. (A–D) Immunohistochemical staining of Arg-1, iNOS, OCN, and RunX2 (Scale bar: 250 μm). (E–H) Quantitative analysis of Arg-1, iNOS, OCN, and RunX2 (n = 3). Statistical significance was determined by One-way ANOVA followed by Bonferroni's multiple comparison test (*P < 0.05, **P < 0.01, and ***P < 0.001).

## 3 Conclusion

### 3.1 Strengths & limitations

This work presents a novel biomaterials approach that combines plant-derived COEVs with a ROS-responsive hydrogel, offering a clear mechanistic focus on macrophage repolarisation and potential relevance to regenerative medicine. Nevertheless, the study lacks long-term in-vivo data, exhibits batch-to-batch variability inherent to plant EV preparations, and does not include a head-to-head comparison with animal-derived EVs.

### 3.2 Bench → clinic → public health

At the bench, we demonstrate that COEVs drive an MMP12/NF-κB axis to tilt the M1/M2 balance in fracture models; whether additional routes such as STAT6 or PI3K/AKT are also engaged awaits future validation. Clinically, the injectable hydrogel provides a biomaterials-based option for orthopaedic cases where conventional grafts or biologics are constrained. From a public-health perspective, scalable Calendula cultivation and low-cost vesicle isolation could democratise musculoskeletal care worldwide—paralleling how accessible, biology-led innovations have been proposed to address global ageing challenges [56].

In conclusion, our findings establish COEV-laden ROS-responsive hydrogels as a promising pre-clinical candidate for fracture repair, while underscoring the need for long-term studies, standardised EV manufacturing, and early regulatory dialogue to advance toward clinical evaluation.

## 4 Materials and methods

### 4.1 Data acquisition

This study obtained datasets from the Gene Expression Omnibus (GEO) database (https://www.ncbi.nlm.nih.gov/geo/), specifically from GSE217792. The data were collected from the intramedullary canal tissue of three patients with nonunion fractures and three normal bone samples.

### 4.2 scRNA-seq data processing and cell type identification

For scRNA-seq analysis, we used the Seurat package [57] to generate a Seurat object from the GSE217792 dataset. Cells with fewer than 500 expressed genes or an elevated mitochondrial gene expression level (>20 %) were excluded. We then selected the top 2000 variable genes using the FindVariableFeatures function and normalized the data. Principal component analysis (PCA) was performed using ScaleData and RunPCA. After sample integration with Harmony, a KNN graph was created using the FindNeighbors function based on the top 30 principal components. Clustering was performed using FindClusters at a resolution of 0.3, and the UMAP method was applied for dimensionality reduction and visualization. Cell types and subtypes were identified based on the expression of known marker genes.

### 4.3 Cell-cell interaction analysis

Intercellular communication, mediated by ligand-receptor interactions, plays a critical role in disease progression. We employed the CellChat tool [58] to analyze differences in cell-cell interactions between normal and fracture samples. By evaluating ligand and receptor gene expression across different cell types, CellChat inferred intercellular connections and identified predominant ligand-receptor interactions. Default parameters were used, and cells with fewer than 10 observations per group were excluded.

### 4.4 Differential expression and functional enrichment analysis

We used the FindMarkers function to identify differentially expressed genes (DEGs) between fracture and normal samples, applying the Wilcoxon test for statistical significance. DEGs were defined based on an absolute log2FC > 1 and p < 0.05. Functional enrichment analysis was conducted using GO and the KEGG databases. GSEA was conducted using the R package clusterProfiler [59], based on the normalized gene expression matrix. The curated gene sets for pathway analysis were retrieved from the Molecular Signatures Database (MSigDB). Enrichment was evaluated using normalized enrichment scores (NES), while significance was determined through adjusted p-values. In addition, GO and KEGG pathway enrichment analyses were applied to the upregulated DEGs, also using the clusterProfiler package to identify significantly overrepresented biological processes and signaling pathways.

### 4.5 Metabolomics analysis of COEVs

To perform untargeted metabolomics analysis, 30 μL of COEVs samples were analyzed using a high-performance liquid chromatography (HPLC) system (Thermo Scientific™ Dionex™ UltiMate™ 3000) coupled with a mass spectrometry system (Q Exactive, Thermo Fisher Scientific). The analysis was supported by data from the Human Metabolome Database (HMDB) and the LIPID MAPS Structure Database (LMSD) to elucidate the metabolite fractions of COEVs.

### 4.6 Identification of Exosomal target components

Potential targets of COEVs compounds were identified using the SwissTargetPrediction (http://swissTargetPrediction.ch/) and GeneCards (https://www.genecards.org/) databases. The overlapping targets from both databases were compiled for further analysis.

### 4.7 Construction of the compound-target network

A compound-target interaction network was constructed using Cytoscape (version 3.9.1) (https://cytoscape.org/).

### 4.8 Materials

Konjac glucomannan (KGM), gelatin derived from porcine skin, 3-carboxybenzeneboronic acid (3-carboxy-BA), N-(3-dimethylaminopropyl)-N′-ethylcarbodiimide hydrochloride (EDC), N-hydroxysuccinimide (NHS), and 2-(N-morpholino) ethanesulfonic acid (MES) buffer were purchased from Aladdin Reagent (Shanghai, China). 1,2-dioleoyl-sn–glycero-3- phosphocholine (DPPC), 1,2-dipalmitoyl-sn–glycero-3- phospho-L–serine and R848 were purchased from Aladdin Reagent, unless otherwise specified, all chemical reagents used in the experiments were sourced from Aladdin Reagent (Shanghai, China) and utilized without further purification. *Calendula officinalis* L. were purchased from the local market.

### 4.9 Isolation and characterization of COEVs

*Calendula officinalis* L., obtained from a market in Guangdong, China, were employed for the extraction of EV-like nanovesicles. The plant material was washed three times with deionized water at 20 °C to eliminate impurities such as dust, soil, and pesticide residues. Following the final wash, *Calendula officinalis* L. was processed into juice using a slow-speed juicer. The juice was subsequently subjected to a series of centrifugation steps at progressively higher speeds (200×*g* for 10 min, 3000×*g* for 20 min, 5000×*g* for 20 min, and 10,000×*g* for 30 min) to remove larger particles and fibers. The supernatant was further purified by ultracentrifugation at 130,000×*g* for 90 min using a Beckman Optima XPN-100 ultracentrifuge (USA) to isolate the nanovesicles. The purified COEVs were stored at −80 °C for subsequent experiments.

### 4.10 Preparation of PSL nanoliposomes

A lipid formulation comprising 6.5 μmol DPPC, 1.5 μmol 1,2-dipalmitoyl-sn-glycero-3-phospho-L-serine, and 2 μmol cholesterol was initially combined in chloroform at 45 °C for 30 min. Subsequent solvent removal via rotary evaporation at 52 °C (2 h) generated uniform thin-film deposits. Hydration with PBS preceded low-temperature ultrasonication (4 °C, 5 min) to yield phosphatidylserine-based liposomes. Terminal size homogenization was achieved through quadruple extrusion across 100 nm polycarbonate membranes, producing monodisperse PSL nanovesicles.

### 4.11 Preparation of PS-COEVs

The specific steps for mixing COEVs and PSL were as follows: first, COEVs (1 mg/mL) and PSL (1 mg/mL) were suspended separately in phosphate buffer solution (PBS, pH 7.4) and briefly vortexed under ice-bath conditions to ensure adequate mixing. Subsequently, an ultrasonic fusion method was employed to facilitate the integration of the two membrane structures. A probe-type ultrasound instrument (power: 25 W; pulse mode: 5 s on/10 s off, repeated 10 times) was used, and an ice bath was consistently maintained to prevent vesicle rupture due to localized overheating. Following ultrasonication, the system underwent 4 °C equilibration (1 h) to facilitate bilayer reconstitution. Terminal purification via 0.22 μm sterile filtration yielded a monodisperse PS-COEVs suspension, cryopreserved at −80 °C for downstream applications. Colloidal properties (hydrodynamic diameter, surface charge) were characterized by dynamic light scattering (Zetasizer), while nanoscale architecture was resolved through TEM.

The purified PS-COEVs sample was placed onto a carbon-coated copper grid, and any excess liquid was removed after adsorption. Subsequently, negative staining was performed using 1 % phosphotungstic acid, followed by gentle blotting and air-drying. The PS-COEVs were then observed and imaged using a transmission electron microscope operated at an acceleration voltage of approximately 100 kV.

The PS-COEVs sample was diluted to a concentration of 2 % (v/v) with deionized water. After loading the diluted sample into a cuvette, measurements were carried out using a laser particle size analyzer maintained at 25 °C to determine the particle size distribution and zeta potential.

### 4.12 Endocytosis of PS-COEVs

To investigate the cellular internalization of PS-COEVs labeled with PKH26, RAW264.7 macrophages were cultured separately with COEVs or PS-COEVs for 24 h. Following incubation, cells underwent sequential fixation with 4 % paraformaldehyde (15 min), permeabilization using 0.1 % Triton X-100 (10 min), and blocking with 5 % BSA (1 h). Cytoskeletal architecture was visualized with Actin Tracker Green (Beyotime, China), while nuclei were counterstained with Hoechst 33342 (Sigma, USA). Subcellular localization of PKH26-labeled PS-COEVs was analyzed using a ZEISS LSM 980 confocal laser microscope (Germany), with fluorescence signals quantified via ZEN imaging software to assess endocytic efficiency.

### 4.13 ROS measurement

Raw264.7 cells were subjected to polarization to M1 type induced by LPS. Dichlorodihydrofluorescein diacetate (DCFH-DA) was diluted to a concentration of 100 μM using serum-free DMEM and added to the treated M1 type macrophages, which were subsequently incubated in darkness for 30 min. Fluorescence was observed using a confocal microscope (ZEISS LSM 980, Germany).

### 4.14 Synthesis and characterization of Gel-BA

Gelatin (3 g) was dissolved in 300 mL of MES buffer solution (pH 5.5) to achieve complete solubilization. Subsequently, 2.9 g of EDC, 0.7 g of NHS, and 1.2 g of 3-carboxy-BA were sequentially added to the gelatin solution. The mixture was then maintained under constant stirring at a controlled temperature of 37 °C for 48 h to facilitate the coupling reaction. Following the reaction, the resulting mixture was purified by dialysis using a dialysis membrane against deionized water (adjusted to pH 7.4) over a period of three days, with six water replacements. Upon completion of dialysis, the final product was subjected to freeze-drying. The physical and chemical properties of the obtained GelBA were characterized and verified using FTIR spectroscopy (Nicolet 6700, Thermo-Fisher, USA) and ^1^H NMR spectroscopy (AVANCE III HD 600 MHz, Bruker, Switzerland).

### 4.15 Preparation and characterization of the gel-BA/KGM/EVs hydrogel

To prepare the Gel-BA/KGM hydrogel, Gel-BA (50 mg) was first dissolved at room temperature in 1 mL of phosphate-buffered saline (PBS) to achieve a final concentration of 5 % w/v. Meanwhile, KGM (15 mg) was dissolved in 1 mL of PBS at 50 °C and placed in a separate centrifuge tube. Subsequently, the KGM solution was mixed with the previously prepared Gel-BA solution, and the pH was adjusted to 7.5–8.5 using a 1 mM sodium hydroxide (NaOH) solution. The mixed solution was gently stirred and kept at room temperature, where the Gel BA/KGM hydrogel rapidly formed. To obtain the Gel BA/KGM/EVs hydrogel, EVs (80 μg) were uniformly dispersed in 1 mL of Gel BA solution and then mixed with an equal volume of KGM solution. The pH was adjusted to 7.5–8.5 using a 1 mM NaOH solution, and the Gel BA/KGM/EVs hydrogel rapidly formed at room temperature. The chemical structure and functional groups of the composite hydrogel were characterized by infrared spectroscopy.

### 4.16 Rheology analysis

The mechanical properties of the hydrogels were assessed using a rotational rheometer (Physica MCR301, Anton Paar) under a strain amplitude of 0.5 %, with frequencies varying from 0.1 to 10 Hz. The injectability of the hydrogels was evaluated by monitoring the variation in linear viscosity (η) as a function of shear rate in frequency scanning mode. Self-healing capabilities were investigated through alternating step-strain sweep tests performed at a constant frequency of 10 rad^−1^. The tests alternated between a low oscillatory strain of 1.0 % and a high oscillatory strain of 300 %, with each strain level maintained for 100 s.

### 4.17 Adhesion tests

The adhesion strength of the hydrogel was evaluated using an electronic mechanical universal testing machine (Sansi Taijie, Zhuhai). The hydrogel solution was applied to two bovine bone slices with an overlapping area of 1 × 1 cm^2^. After gelation, the assembled samples were subjected to lap-shear testing at a crosshead speed of 7 mm/min.

### 4.18 Responsive Degradability

The responsiveness of the hydrogels to external stimuli was assessed by subjecting them to different conditions. Equal volumes of hydrogel were placed in centrifuge tubes, to which 100 μL of PBS solution, hydrochloric acid (HCl, pH = 5.0), and hydrogen peroxide (H_2_O_2_) were added separately. The mixtures were then incubated at 37 °C for 36 h. The release of PS-COEVs was assessed using fluorescent labeling. DID was employed to label the PS-COEVs, and the fluorescence signal was measured with a fluorescent microplate reader. The cumulative release rate of PS-COEVs was calculated using the following formula:

$$Cumulative\;release\;\left(\%\right)=\frac{F_{t}-F_{0}}{F_{\max}-F_{0}}\times 100$$

Where *F*_*t*_ represents the fluorescence value of the medium at time point *t*, *F*_*0*_ denotes the initial fluorescence value of the medium, and *F*_max_ refers to the maximum fluorescence value.

### 4.19 Swelling tests

The swelling rate (SRs) of the hydrogel was determined using the immersion method. A pre-weighed hydrogel sample (2.7 g, W_0_) was immersed in PBS for a designated period. Afterward, the swollen hydrogel was retrieved, and surface water was carefully removed before recording its wet weight (W_t_). The swelling rate was calculated using the formula: SR_s_ = [(W_t_ − W_0_)/W_0_] × 100 %.

### 4.20 Cell culture

The RAW264.7 macrophage cell line (American Type Culture Collection, ATCC, Manassas, VA, USA) was cultured in high-glucose Dulbecco's Modified Eagle Medium (DMEM) supplemented with 10 % fetal bovine serum (FBS), 1 % penicillin-streptomycin, and 1 % glutamine. Bone marrow-derived mesenchymal stem cells (BMSCs) were isolated from the bilateral humeri and femora of 2-week-old female Sprague Dawley (SD) rats via bone marrow flushing and subsequently cultured in low-glucose DMEM containing 10 % FBS (Gibco, USA). For the co-culture of hydrogel and cells, the hydrogel premix was initially introduced into the well plate to form a gel. Subsequently, the gel was sterilized through a series of immersion processes: 12 h in 75 % ethanol, followed by 1 h in PBS, 12 h in a 10 % double-antibody solution, and a final 1-h immersion in PBS. After sterilization, cells of an appropriate density were seeded into the well plate. All cells were maintained at 37 °C in a humidified incubator with 5 % CO_2_.

### 4.21 In vitro biocompatibility testing

Biocompatibility was evaluated by assessing cell viability, proliferation, and adhesion. BMSCs (1 × 10^6^ cells/well) were co-cultured with test materials for 24 h. For M1 polarization macrophages, RAW 264.7 cells were treated with 100 ng/mL LPS for 24 h and M1 polarization macrophages (1 × 10^6^ cells/well) were co-cultured with test materials for 24 h. Cell viability was determined using Calcein AM/Propidium Iodide (Calcein AM/PI, Invitrogen, USA). After 3 days of co-culture, 100 μL of Cell Counting Kit-8 (CCK-8, Beyotime, China) solution was added to each well. Following a 3-h incubation, supernatants were transferred to 96-well plates, and absorbance was measured at 450 nm using a spectrophotometer (BioTech, China). Cell adhesion and spreading were analyzed by staining the cytoskeleton with Actin-Tracker Green (Beyotime). Five random fields per sample were imaged, and cell spreading areas were quantified using ImageJ software (NIH, USA).

### 4.22 Hemolysis test

Fresh rat whole blood was diluted with saline and incubated with Gel-BA/KGM@PS-COEVs at 37 °C for 2 h. The mixture was centrifuged at 3000 rpm for 10 min, and the absorbance of the supernatant was measured at 545 nm using a microplate reader (MK3, Thermo Fisher, USA). Negative and positive controls were prepared using 2 % Triton X-100 and phosphate-buffered saline (PBS), respectively.

### 4.23 PS-COEVs Biodistribution

For in vivo fluorescence imaging, rat was subcutaneous injection with Gel-BA/KGM@PS-COEVs. After injections for 0,1,3,5,7 days, the rat was sacrificed and major tissues (liver, heart, spleen, kidney and lung) were isolated and imaged.

### 4.24 In vivo Compatibility assessment of gel-BA/KGM@PS-COEVs

Gel-BA/KGM@PS-COEVs were administered to rat (treatment group), while the control group received PBS. Blood samples were collected 7 days post-injection to analyze liver/kidney function biomarkers and hematological parameters. Major organs (heart, lungs, liver, kidneys, spleen) were harvested for histopathological examination using hematoxylin and eosin (H&E) staining.

### 4.25 Gene expression analysis

For the in-vitro M1→M2 assay, RAW264.7 cells were treated and harvested at 24 h post-stimulation. Total RNA was extracted using an RNA isolation kit (Omega Bio-Tek, USA) and reverse-transcribed into cDNA (Takara, Japan). RT-qPCR was performed with LightCycler 480 SYBR Green Master Mix (Takara). M1 markers (iNOS, TNF-α) and M2 markers (Arg-1, IL-10) were normalized to GAPDH and calculated with the 2^−ΔΔCt^ method (primers listed in Table S2; triplicates per group).

### 4.26 Immunofluorescence staining

After 24 h of treatment in the M1→M2 assay, cells were fixed with 4 % paraformaldehyde (30 min), permeabilized with 0.2 % Triton X-100 (Beyotime, 1h), and blocked with 3 % bovine serum albumin (BSA, Beyotime, 1h). Primary antibodies against iNOS (M1 marker) and Arg-1 (M2 marker) (Table S1) were applied overnight at 4 °C, followed by 1 h incubation with secondary antibodies at room temperature. Nuclei were counterstained with Hoechst 33342 (Sigma-Aldrich, USA), and images were acquired using a confocal microscope (LSM 980, ZEISS, Germany).

### 4.27 WB assay

Whole-cell lysates were prepared at 24 h of the M1→M2 experiment. Cells were lysed in RIPA buffer (CWBIO, China) containing protease/phosphatase inhibitors (Thermo Fisher). Lysates were sonicated, centrifuged, and quantified using a BCA assay kit (Beyotime). Proteins (40 μg/lane) were separated by SDS-PAGE (Beyotime) and transferred to PVDF membranes (Thermo Fisher). After blocking with High-Efficiency Blocking Solution (Genefist, China), the membranes were incubated with primary antibodies against iNOS (M1) and Arg-1 (M2) (Table S1) and then visualized with an ECL kit (Thermo Fisher). Band intensities were analyzed via ImageJ.

### 4.28 ELISA assay

Supernatants from the 24-h M1→M2 assay were centrifuged (300×*g*, 5 min, 4 °C) and stored at −80 °C. TNF-α (M1) and IL-10 (M2) levels were measured in duplicate using Beyotime sandwich ELISA kits (cat. PI523 & PT513). Pre-coated 96-well plates were incubated overnight with 100 μL undiluted sample at 4 °C, followed by biotinylated detection antibody (2 h, RT), streptavidin-HRP (30 min) and TMB substrate (15 min), and absorbance read at 450 nm on a microplate reader.

### 4.29 Flow cytometry assay

At 24 h post-treatment in the M1→M2 experiment, RAW264.7 cells were treated as indicated, detached with enzyme-free dissociation buffer, washed twice in ice-cold PBS. After blocking 10 min, 4 °C, cells were incubated with fluorochrome-conjugated antibodies: CD11b-FITC (Biolegend, cat.101205), CD86-PE (Biolegend, cat. 159203) and CD206-APC (Biolegend, cat. 141707) for 30 min on ice, protected from light. Following three washes, samples were acquired on a BD FACSLyric flow cytometer (BD Biosciences, USA) using unstained and single-stained compensation controls. Data were analyzed with FlowJo v10.8 (BD). At least 10,000 viable CD11b^+^ events were collected per replicate, and the percentages of CD86^+^ (M1) and CD206^+^ (M2) populations were calculated.

### 4.30 Osteoimmunomodulatory effects on osteogenic differentiation

A Transwell co-culture system was used to evaluate macrophage-mediated osteogenesis. BMSCs (1 × 10^4^ cells) were seeded in the upper chamber, while RAW264.7 macrophages (1 × 10^5^ cells) were placed in the lower chamber, separated by a 1.0 μm pore polycarbonate membrane. This setup allowed cytokine exchange under Gel-BA/KGM@PS-COEVs treatment.

### 4.31 Staining and quantification of ALP

BMSCs were fixed with 4 % paraformaldehyde (30 min) and stained using an ALP Staining Kit (Beyotime). After 1 h, samples were imaged under an optical microscope (Olympus, Japan). ALP activity was quantified using an ALP Assay Kit (Beyotime) at 450 nm (BioTech microplate reader) following 7 days of osteogenic induction.

### 4.32 ARS staining and quantification

Mineralized nodules were stained with 2 % Alizarin Red S (Sigma) for 10 min and imaged (Canon DSLR). For quantification, stained samples were dissolved in 10 % sodium phosphate/5 % cetylpyridinium chloride (Sigma), and absorbance was measured at 570 nm.

### 4.33 Femoral fracture model

To investigate the effects of Gel-BA/KGM@PS-COEVs on femoral fractures, 6-week-old female SD rats were employed, all animal experiments were submitted for review to the Animal Ethics (2020DZGZRZX-078). A mid-femoral fracture model was created using a rongeur after exposing the femur. The fracture ends were stabilized with 1.2 mm diameter Kirschner wires, and approximately 15 μL of Gel-BA/KGM@PS-COEVs was applied to bind the fragments. A total of 24 rats were randomly divided into four groups: Control group (n = 6), Gel-BA/KGM group (n = 6), COEVs group (n = 6), and Gel-BA/KGM@PS-COEVs group (n = 6). All rats were sacrificed after 8 weeks.

### 4.34 Micro-CT observation

Micro-CT imaging was conducted using a Scanco mCT50 (Switzerland) to scan the femurs. To assess bone healing and the biodegradation of the implants, 3D reconstructions were generated using Materialize Mimics Research 19. Regions of interest (ROIs) were delineated within 0.5 mm of the intramedullary nails. Quantitative analyses were performed on these ROIs, encompassing measurements of BMD, BV/TV, and Tb.Th.

### 4.35 Histological analysis and immunohistochemistry

After 8 weeks of treatment, the entire femurs of the rats were collected. Following fixation in 4 % paraformaldehyde, the rat femurs were decalcified in a decalcifying solution for 21 days. A 5 mm section was cut from each specimen and embedded in paraffin. The femoral fracture site was stained with hematoxylin and eosin (H&E) and Masson's trichrome, in addition to immunohistochemistry assays. Antibodies used for immunohistochemistry included *anti*-iNOS, *anti*-Arg-1, *anti*-OCN, and *anti*-RunX2 (Table S2).

### 4.36 Statistical analysis

Normality and homogeneity. All continuous variables were first tested for normality with the Shapiro–Wilk test and for equal variances with Levene's test (P > 0.05 indicated parametric suitability). Two-group comparisons. When only two independent groups were compared (e.g., Control vs. Gel-BA/KGM@PS-COEVs), an unpaired two-tailed Student's t-test was applied if data were normally distributed; otherwise, the Mann–Whitney *U* test was used. Multi-group comparisons. For single-factor designs with more than two groups (Control, Gel-BA/KGM, PS-COEVs, Gel-BA/KGM@PS-COEVs), one-way ANOVA was employed when normality and homogeneity were met, followed by Tukey's post-hoc test for pairwise comparisons with family-wise error control (adjusted α = 0.05). When either assumption was violated, the Kruskal–Wallis test with Dunn's multiple-comparison correction (adjusted α = 0.05) was performed. All P-values are reported as two-tailed exact values; P < 0.05 was deemed statistically significant. Software. Analyses were conducted in GraphPad Prism v9.5.1.

## CRediT authorship contribution statement

**Xuesong Wang:** Writing – original draft, Validation, Methodology, Investigation, Formal analysis. **Zikun Xie:** Writing – original draft, Validation, Methodology, Investigation, Formal analysis. **Riyue Wu:** Validation, Methodology, Investigation. **Pengcheng Gao:** Validation, Methodology, Investigation. **Liqin Chen:** Investigation. **Junlin Zhong:** Writing – review & editing, Writing – original draft, Supervision, Project administration, Funding acquisition, Data curation, Conceptualization. **Shuofei Yang:** Writing – review & editing, Supervision, Project administration, Funding acquisition, Data curation, Conceptualization. **Lei Jin:** Writing – review & editing, Supervision, Project administration, Funding acquisition, Data curation, Conceptualization.

## Ethics approval and consent to participate

Animal experimental protocols were approved by the Jinling Hospital of Nanjing University (2020DZGZRZX-078).

## Declaration of competing interest

The authors declare that they have no known competing financial interests or personal relationships that could have appeared to influence the work reported in this paper.

## Figures and Tables

**figs1:**
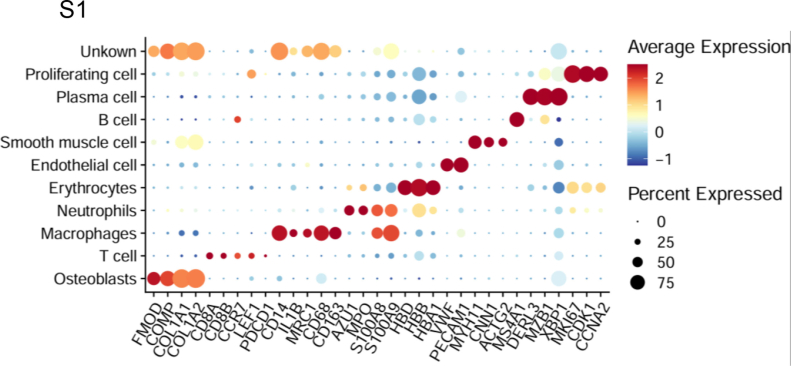


**figs2:**
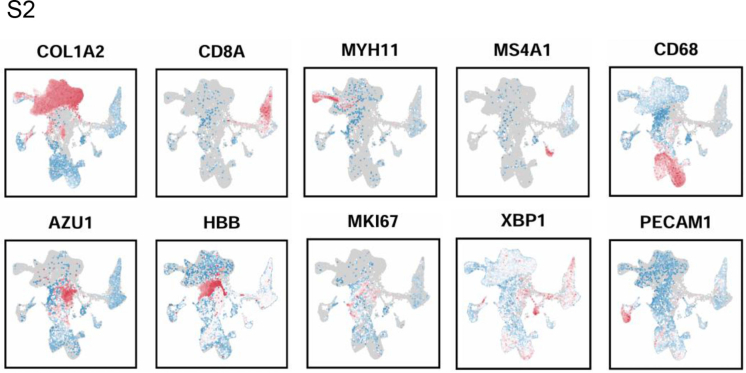


**figs3:**
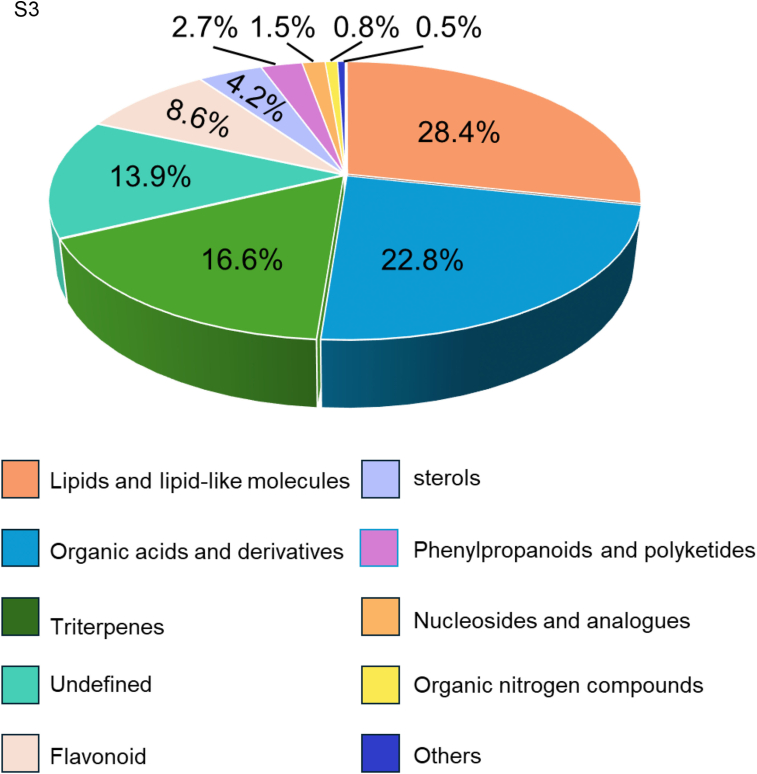


**figs4:**
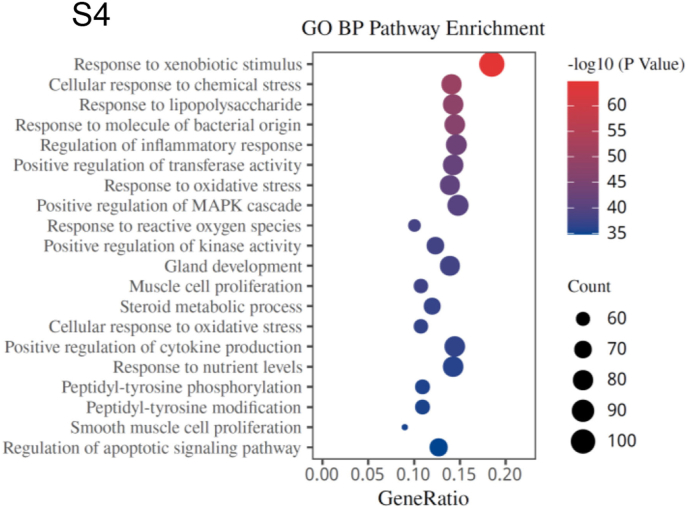


**figs5:**
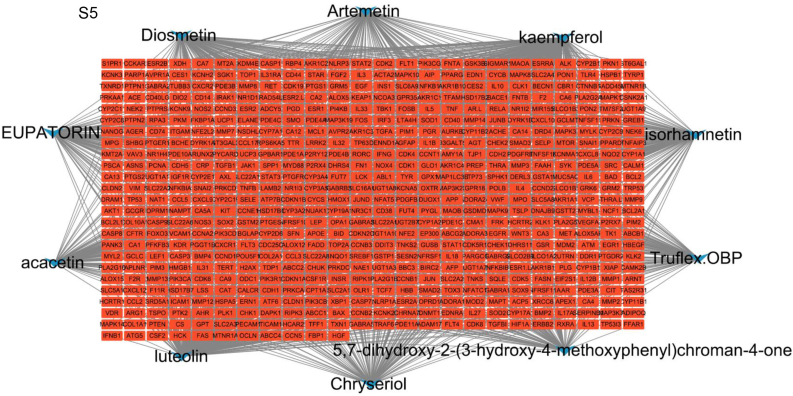


**figs6:**
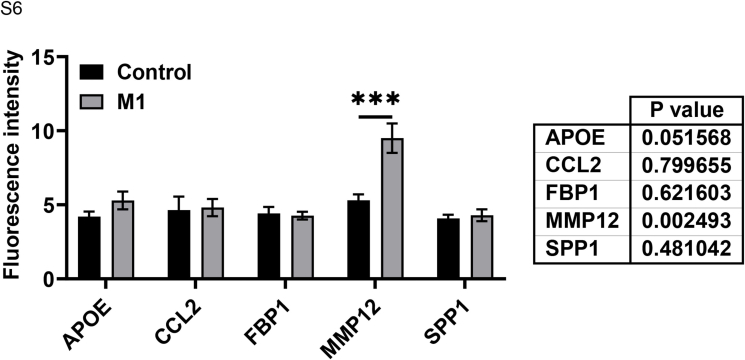


**figs7:**
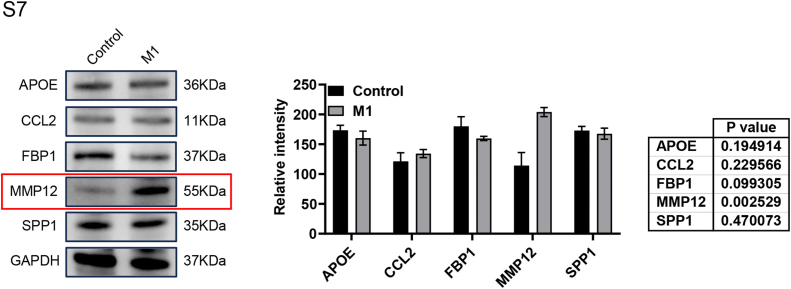


**figs8:**
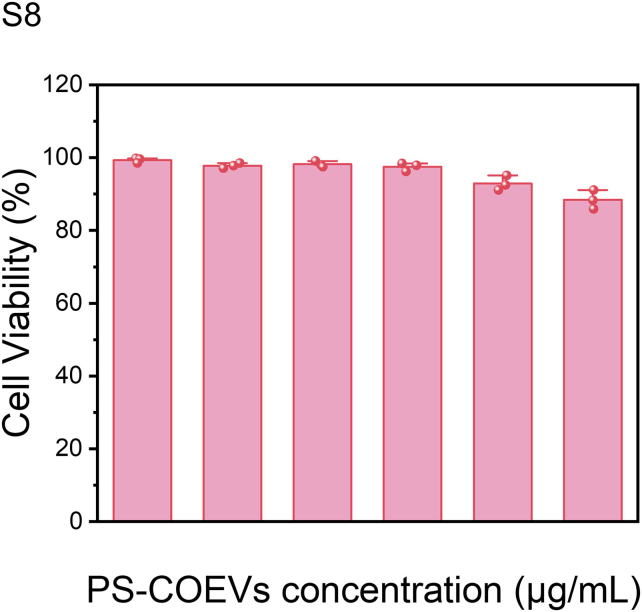


**figs9:**
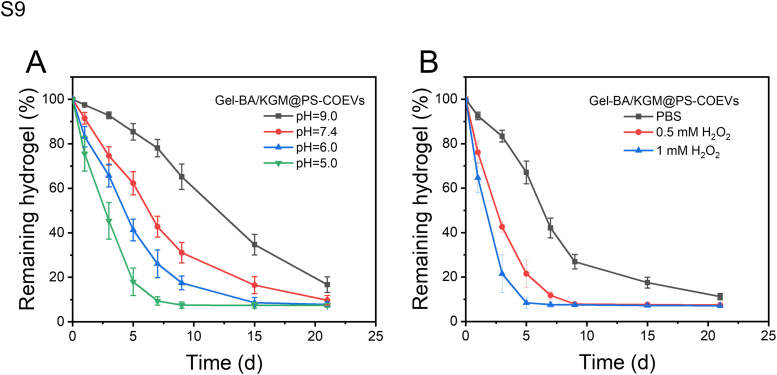


**figs10:**
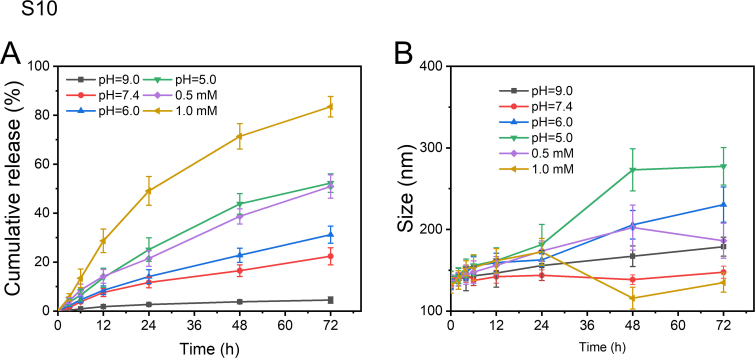


**figs11:**
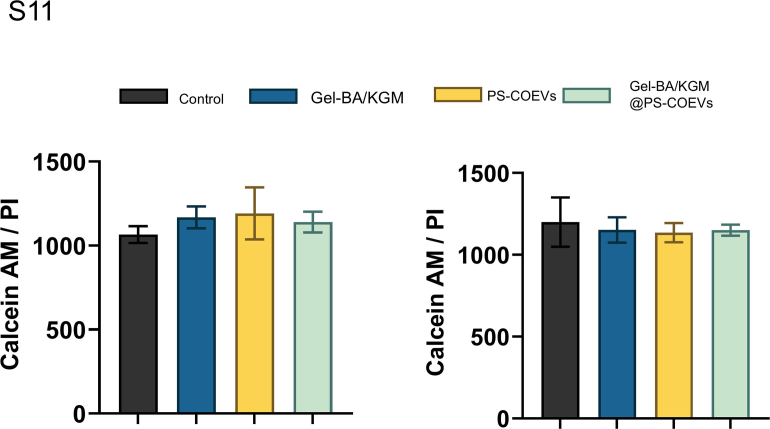


**figs12:**
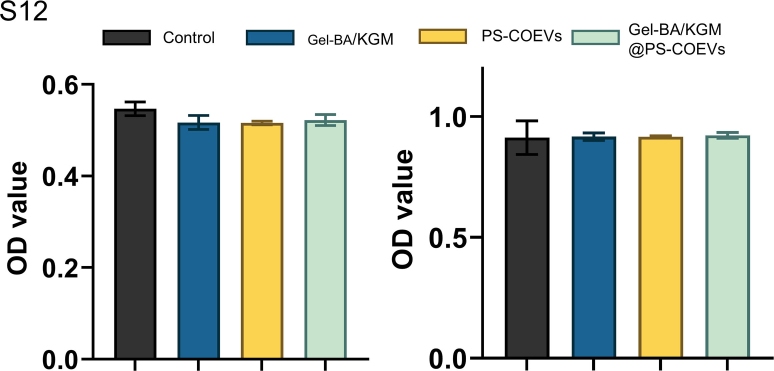


**figs13:**
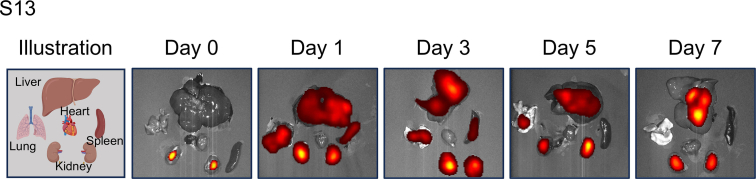


**figs14:**
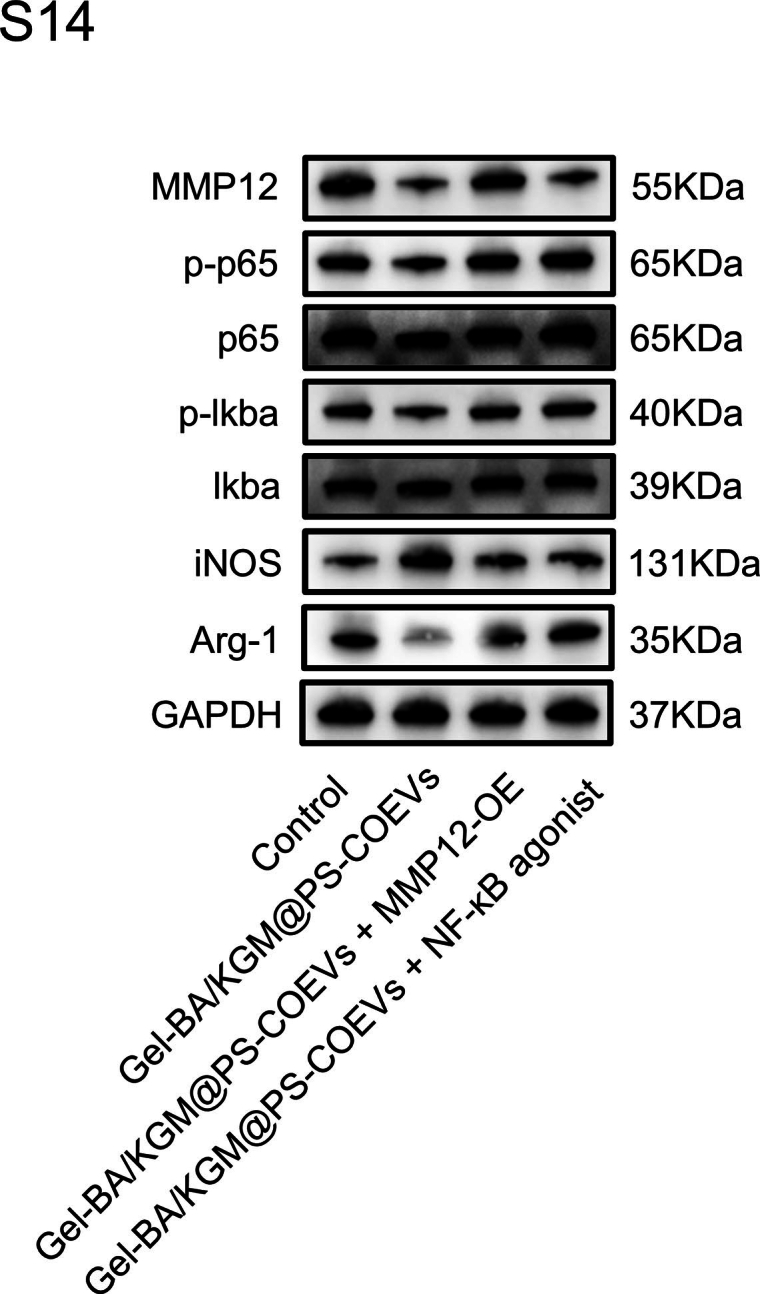


**figs15:**
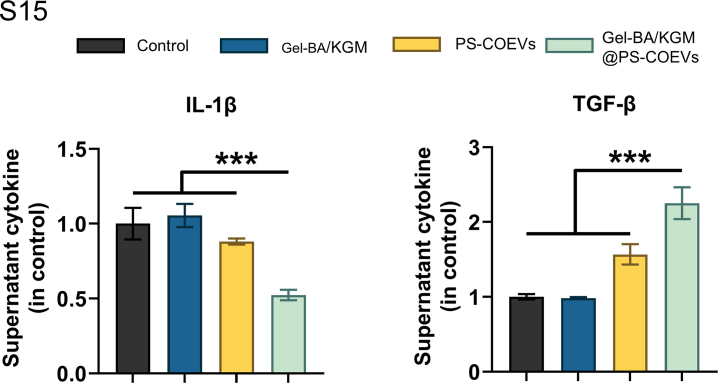


**figs16:**
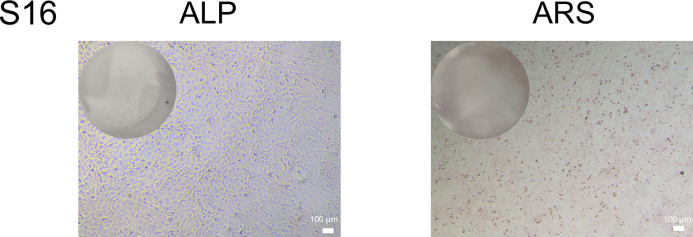


**figs17:**
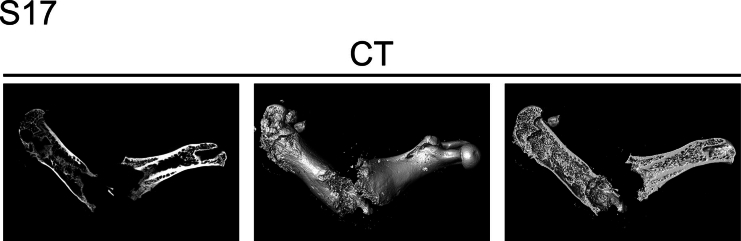


**figs18:**
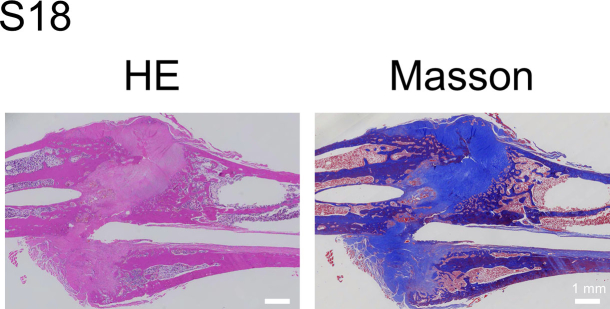


**figs19:**
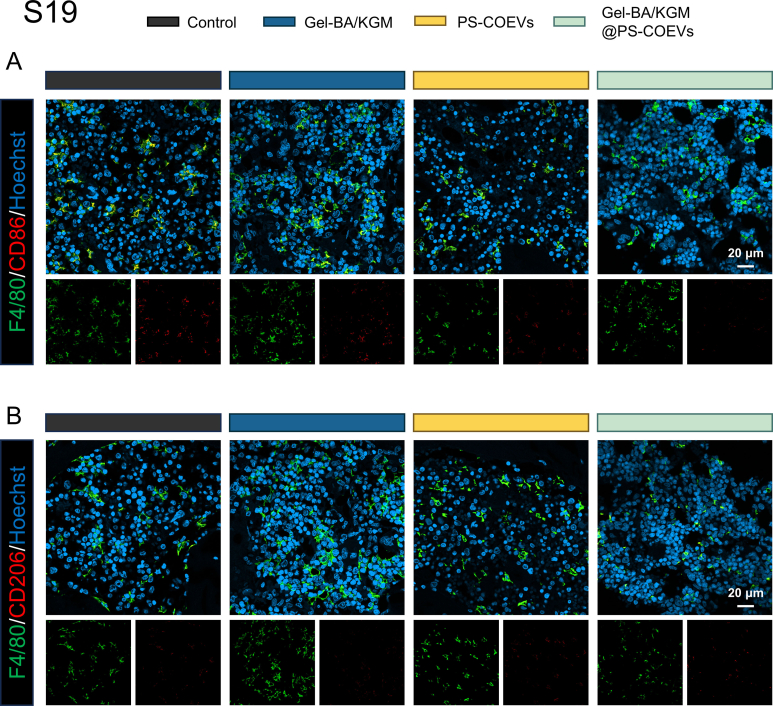


**figs20:**
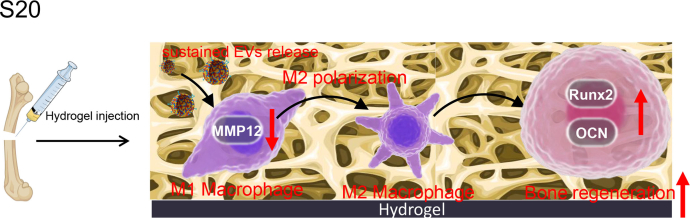


## Data Availability

Data will be made available on request.

## References

[1] Zhang M., Xu F., Cao J., Dou Q., Wang J., Wang J., Yang L., Chen W. (2024). Research advances of nanomaterials for the acceleration of fracture healing. Bioact. Mater..

[2] Wildemann B., Ignatius A., Leung F., Taitsman L.A., Smith R.M., Pesántez R., Stoddart M.J., Richards R.G., Jupiter J.B. (2021). Non-union bone fractures. Nat. Rev. Dis. Primers.

[3] Ho-Shui-Ling A., Bolander J., Rustom L.E., Johnson A.W., Luyten F.P., Picart C. (2018). Bone regeneration strategies: engineered scaffolds, bioactive molecules and stem cells current stage and future perspectives. Biomaterials.

[4] Ahmad M., Krüger B.T., Kroll T., Vettorazzi S., Dorn A.-K., Mengele F., Lee S., Nandi S., Yilmaz D., Stolz M., Tangudu N.K., Vázquez D.C., Pachmayr J., Cirstea I.C., Spasic M.V., Ploubidou A., Ignatius A., Tuckermann J. (2022). Inhibition of Cdk5 increases osteoblast differentiation and bone mass and improves fracture healing. Bone Res..

[5] Wang S., Qiu J., Guo A., Ren R., He W., Liu S., Liu Y. (2020). Nanoscale perfluorocarbon expediates bone fracture healing through selectively activating osteoblastic differentiation and functions. J. Nanobiotechnol..

[6] Chen M., Wang D., Li M., He Y., He T., Chen M., Hu Y., Luo Z., Cai K. (2022). Nanocatalytic biofunctional MOF coating on titanium implants promotes osteoporotic bone regeneration through cooperative pro-osteoblastogenesis MSC reprogramming. ACS Nano.

[7] Zhu Y., Liu H., Wu P., Chen Y., Deng Z., Cai L., Wu M. (2024). Multifunctional injectable hydrogel system as a mild photothermal-assisted therapeutic platform for programmed regulation of inflammation and osteo-microenvironment for enhanced healing of diabetic bone defects in situ. Theranostics.

[8] Ye S., Cao Q., Ni P., Xiong S., Zhong M., Yuan T., Shan J., Liang J., Fan Y., Zhang X. (2025). A ceramic microbridge microfluidic chip to study osteogenic differentiation of mesenchymal stem cells in bioactive ceramic immune microenvironment. Bioact. Mater..

[9] Zhang F., Lv M., Wang S., Li M., Wang Y., Hu C., Hu W., Wang X., Wang X., Liu Z., Fan Z., Du J., Sun Y. (2024). Ultrasound-triggered biomimetic ultrashort peptide nanofiber hydrogels promote bone regeneration by modulating macrophage and the osteogenic immune microenvironment. Bioact. Mater..

[10] Cai Z., Bai L., Li Q., Li Y., Cai X., Lin Y. (2024). Gene-activating framework nucleic acid-targeted upregulating Sirtuin-1 to modulate osteoimmune microenvironment for diabetic osteoporosis therapeutics. ACS Nano.

[11] Yu Z., Wang Z., Chen Y., Wang Y., Tang L., Xi Y., Lai K., Zhang Q., Li S., Xu D., Tian A., Wu M., Wang Y., Yang G., Gao C., Huang T. (2025). Programmed surface platform orchestrates anti-bacterial ability and time-sequential bone healing for implant-associated infection. Biomaterials.

[12] Wang D., Chen M.-W., Wei Y.-J., Geng W.-B., Hu Y., Luo Z., Cai K.-Y. (2022). Construction of wogonin nanoparticle-containing strontium-doped nanoporous structure on titanium surface to promote osteoporosis fracture repair. Adv. Healthcare Mater..

[13] Qin D., Zhao Y., Cheng R., Liu Y., Guo S., Sun L., Guo Y., Hao F., Zhao B. (2024). Mussel-inspired immunomodulatory and osteoinductive dual-functional hydroxyapatite nanoplatform for promoting bone regeneration. J. Nanobiotechnol..

[14] Wu H., Dong H., Tang Z., Chen Y., Liu Y., Wang M., Wei X., Wang N., Bao S., Yu D., Wu Z., Yang Z., Li X., Guo Z., Shi L. (2023). Electrical stimulation of piezoelectric BaTiO3 coated Ti6Al4V scaffolds promotes anti-inflammatory polarization of macrophages and bone repair via MAPK/JNK inhibition and OXPHOS activation. Biomaterials.

[15] Zhao Y., Bai L., Zhang Y., Yao R., Sun Y., Hang R., Chen X., Wang H., Yao X., Xiao Y., Hang R. (2022). Type I collagen decorated nanoporous network on titanium implant surface promotes osseointegration through mediating immunomodulation, angiogenesis, and osteogenesis. Biomaterials.

[16] Zhang Y., Wang D., Peng M., Tang L., Ouyang J., Xiong F., Guo C., Tang Y., Zhou Y., Liao Q., Wu X., Wang H., Yu J., Li Y., Li X., Li G., Zeng Z., Tan Y., Xiong W. (2021). Single-cell RNA sequencing in cancer research. J. Exp. Clin. Cancer Res..

[17] Li X., Wang C.-Y. (2021). From bulk, single-cell to spatial RNA sequencing. Int. J. Oral Sci..

[18] Van de Sande B., Lee J.S., Mutasa-Gottgens E., Naughton B., Bacon W., Manning J., Wang Y., Pollard J., Mendez M., Hill J., Kumar N., Cao X., Chen X., Khaladkar M., Wen J., Leach A., Ferran E. (2023). Applications of single-cell RNA sequencing in drug discovery and development. Nat. Rev. Drug Discov..

[19] Chen H., Ye F., Guo G. (2019). Revolutionizing immunology with single-cell RNA sequencing. Cell. Mol. Immunol..

[20] Liu W., Jia J., Dai Y., Chen W., Pei G., Yan Q., Zhao Z. (2022). Delineating COVID-19 immunological features using single-cell RNA sequencing. Innov.

[21] Deleersnijder D., Callemeyn J., Arijs I., Naesens M., Van Craenenbroeck A.H., Lambrechts D., Sprangers B. (2021). Current methodological challenges of single-cell and single-nucleus RNA-sequencing in glomerular diseases. J. Am. Soc. Nephrol..

[22] Wang M., Song W.-M., Ming C., Wang Q., Zhou X., Xu P., Krek A., Yoon Y., Ho L., Orr M.E., Yuan G.-C., Zhang B. (2022). Guidelines for bioinformatics of single-cell sequencing data analysis in Alzheimer's disease: review, recommendation, implementation and application. Mol. Neurodegener..

[23] Lian M.Q., Chng W.H., Liang J., Yeo H.Q., Lee C.K., Belaid M., Tollemeto M., Wacker M.G., Czarny B., Pastorin G. (2022). Plant-derived extracellular vesicles: recent advancements and current challenges on their use for biomedical applications. J. Extracell. Vesicles.

[24] Zhu Y., Zhao J., Ding H., Qiu M., Xue L., Ge D., Wen G., Ren H., Li P., Wang J. (2024). Applications of plant-derived extracellular vesicles in medicine. MedComm.

[25] Xu Z., Xu Y., Zhang K., Liu Y., Liang Q., Thakur A., Liu W., Yan Y. (2023). Plant-derived extracellular vesicles (PDEVs) in nanomedicine for human disease and therapeutic modalities. J. Nanobiotechnol..

[26] Feng J., Xiu Q., Huang Y., Troyer Z., Li B., Zheng L. (2023). Plant-derived vesicle-like nanoparticles as promising biotherapeutic tools: present and future. Adv. Mater..

[27] Li A., Li D., Gu Y., Liu R., Tang X., Zhao Y., Qi F., Wei J., Liu J. (2023). Plant-derived nanovesicles: further exploration of biomedical function and application potential. Acta Pharm. Sin. B.

[28] Zhu Y., Ouyang Z., Du H., Wang M., Wang J., Sun H., Kong L., Xu Q., Ma H., Sun Y. (2022). New opportunities and challenges of natural products research: when target identification meets single-cell multiomics. Acta Pharm. Sin. B.

[29] Shahane K., Kshirsagar M., Tambe S., Jain D., Rout S., Ferreira M.K.M., Mali S., Amin P., Srivastav P.P., Cruz J., Lima R.R. (2023). An updated review on the multifaceted therapeutic potential of Calendula officinalis L. Pharmaceuticals.

[30] Zhao B., Lin H., Jiang X., Li W., Gao Y., Li M., Yu Y., Chen N., Gao J. (2024). Exosome-like nanoparticles derived from fruits, vegetables, and herbs: innovative strategies of therapeutic and drug delivery. Theranostics.

[31] Kang S.J., Lee J.H., Rhee W.J. (2024). Engineered plant-derived extracellular vesicles for targeted regulation and treatment of colitis-associated inflammation. Theranostics.

[32] Zhuang X., Deng Z.-B., Mu J., Zhang L., Yan J., Miller D., Feng W., McClain C.J., Zhang H.-G. (2015). Ginger-derived nanoparticles protect against alcohol-induced liver damage. J. Extracell. Vesicles.

[33] Khouchlaa A., El Baaboua A., El Moudden H., Lakhdar F., Bakrim S., El Menyiy N., Belmehdi O., Harhar H., El Omari N., Balahbib A., Park M.N., Zengin G., Kim B., Bouyahya A. (2023). Traditional uses, bioactive compounds, and pharmacological investigations of Calendula arvensis L.: a comprehensive review. Adv. Pharmacol. Pharm. Sci..

[34] Di Lorenzo C., Dell'Agli M., Badea M., Dima L., Colombo E., Sangiovanni E., Restani P., Bosisio E. (2013). Plant food supplements with anti-inflammatory properties: a systematic review (II). Crit. Rev. Food Sci. Nutr..

[35] Carvalho A.R.J., Diniz R.M., Suarez M.A.M., Figueiredo C.S.S.E.S., Zagmignan A., Grisotto M.A.G., Fernandes E.S., da Silva L.C.N. (2018). Use of some asteraceae plants for the treatment of wounds: from ethnopharmacological studies to scientific evidences. Front. Pharmacol..

[36] Shen Q., Ge L., Lu W., Wu H., Zhang L., Xu J., Tang O., Muhammad I., Zheng J., Wu Y., Wang S.-W., Zeng X.-X., Xue J., Cheng K. (2024). Transplanting network pharmacology technology into food science research: a comprehensive review on uncovering food-sourced functional factors and their health benefits. Compr. Rev. Food Sci. Food Saf..

[37] Nogales C., Mamdouh Z.M., List M., Kiel C., Casas A.I., Schmidt H.H.H.W. (2022). Network pharmacology: curing causal mechanisms instead of treating symptoms. Trends Pharmacol. Sci..

[38] Xu H., Zhang Y., Wang P., Zhang J., Chen H., Zhang L., Du X., Zhao C., Wu D., Liu F., Yang H., Liu C. (2021). A comprehensive review of integrative pharmacology-based investigation: a paradigm shift in traditional Chinese medicine. Acta Pharm. Sin. B.

[39] Bai C., Liu J., Zhang X., Li Y., Qin Q., Song H., Yuan C., Huang Z. (2024). Research status and challenges of plant-derived exosome-like nanoparticles. Biomed. Pharmacother..

[40] Ding H., Tan P., Fu S., Tian X., Zhang H., Ma X., Gu Z., Luo K. (2022). Preparation and application of pH-responsive drug delivery systems. J. Control. Release Off. J. Control. Release Soc..

[41] Yin P., Liang W., Han B., Yang Y., Sun D., Qu X., Hai Y., Luo D. (2024). Hydrogel and nanomedicine-based multimodal therapeutic strategies for spinal cord injury. Small Methods.

[42] Li Y., Yang H.Y., Lee D.S. (2021). Advances in biodegradable and injectable hydrogels for biomedical applications. J. Control. Release Off. J. Control. Release Soc..

[43] Zheng J., Song X., Yang Z., Yin C., Luo W., Yin C., Ni Y., Wang Y., Zhang Y. (2022). Self-assembly hydrogels of therapeutic agents for local drug delivery. J. Control. Release Off. J. Control. Release Soc..

[44] Sun H., Xu J., Wang Y., Shen S., Xu X., Zhang L., Jiang Q. (2023). Bone microenvironment regulative hydrogels with ROS scavenging and prolonged oxygen-generating for enhancing bone repair. Bioact. Mater..

[45] Xiu H., Yang K., Dong L., Lai H., Zhu Z., Jiang D., Yan J., Shi C., Pan S., Yin Z., Yuwen L., Liang B. (2025). Near-infrared light-responsive Cu(2)MoS(4)@GelMA hydrogel with photothermal therapy, antibacterial effect and bone immunomodulation for accelerating infection elimination and fracture healing. Adv. Healthcare Mater..

[46] Chen M., Sun Y., Hou Y., Luo Z., Li M., Wei Y., Chen M., Tan L., Cai K., Hu Y. (2022). Constructions of ROS-Responsive titanium-hydroxyapatite implant for mesenchymal stem cell recruitment in peri-implant space and bone formation in osteoporosis microenvironment. Bioact. Mater..

[47] Rui M., Mao J., Wu H., Hui Y., Shen H., Yang Y., Ma T., Ren K., Wang J., Cui W., Shi Q., Yang H. (2025). Implantable multifunctional micro-oxygen reservoir system for promoting vascular-osteogenesis via remodeling regenerative microenvironment. Adv. Sci. (Weinheim, Baden-Wurttemberg, Ger..

[48] Scott-Hewitt N., Perrucci F., Morini R., Erreni M., Mahoney M., Witkowska A., Carey A., Faggiani E., Schuetz L.T., Mason S., Tamborini M., Bizzotto M., Passoni L., Filipello F., Jahn R., Stevens B., Matteoli M. (2020). Local externalization of phosphatidylserine mediates developmental synaptic pruning by microglia. EMBO J..

[49] Tian L., Tan Z., Yang Y., Liu S., Yang Q., Tu Y., Chen J., Guan H., Fan L., Yu B., Chen X., Hu Y. (2023). In situ sprayed hydrogels containing resiquimod-loaded liposomes reduce chronic osteomyelitis recurrence by intracellular bacteria clearance. Acta Biomater..

[50] Ehlers M.R. (2000). CR3: a general purpose adhesion-recognition receptor essential for innate immunity. Microbes Infect.

[51] Aderem A., Underhill D.M. (1999). Mechanisms of phagocytosis in macrophages. Annu. Rev. Immunol..

[52] Qian K., Zhou J., Miao M., Thaiboonrod S., Fang J., Feng X. (2024). Stretchable supramolecular hydrogel with instantaneous self-healing for electromagnetic interference shielding control and sensing. Compos. Part B Eng..

[53] Loebel C., Rodell C.B., Chen M.H., Burdick J.A. (2017). Shear-thinning and self-healing hydrogels as injectable therapeutics and for 3D-printing. Nat. Protoc..

[54] Yu C., Qiu Y., Yao F., Wang C., Li J. (2024). Chemically programmed hydrogels for spatiotemporal modulation of the cardiac pathological microenvironment. Adv. Mater..

[55] Chen S., Lu Z., Zhao Y., Xia L., Liu C., Zuo S., Jin M., Jia H., Li S., Zhang S., Yang B., Wang Z., Li J., Wang F., Yang C. (2024). Myeloid-Mas signaling modulates pathogenic crosstalk among MYC(+)CD63(+) endothelial cells, MMP12(+) macrophages, and monocytes in acetaminophen-induced liver injury. Adv. Sci. (Weinheim, Baden-Wurttemberg, Ger..

[56] Chu C. (2025). Burden of dementia: oral health needs attention. BMJ.

[57] Satija R., Farrell J.A., Gennert D., Schier A.F., Regev A. (2015). Spatial reconstruction of single-cell gene expression data. Nat. Biotechnol..

[58] Jin S., Guerrero-Juarez C.F., Zhang L., Chang I., Ramos R., Kuan C.-H., Myung P., V Plikus M., Nie Q. (2021). Inference and analysis of cell-cell communication using CellChat. Nat. Commun..

[59] Wu T., Hu E., Xu S., Chen M., Guo P., Dai Z., Feng T., Zhou L., Tang W., Zhan L., Fu X., Liu S., Bo X., Yu G. (2021). clusterProfiler 4.0: a universal enrichment tool for interpreting omics data. Innov.

