# Supplementary material for: Hydrogel-Wrapped Calendula officinalis L. extracellular vesicles - A novel approach to enhance fracture healing by Macrophage reprogramming
Source: Mater Today Bio. 2025 Nov 28;35:102592. doi: 10.1016/j.mtbio.2025.102592 (PMC12704079; doi:10.1016/j.mtbio.2025.102592)
Supplement: Multimedia component 1 [file mmc1.docx]

**Table S1.** Relative information about primary antibodies

| Catalog Number | Antibodies | Species | Dilution (cell/tissue) | Molecular Weight | Source |
| --- | --- | --- | --- | --- | --- |
| ab8245 | Anti-GAPDH | Mouse | 1:200 | 36kDa | Abcam, England |
| ab 178945 | Anti-iNOS | Rabbit | WB 1:1000  IF and IHC 1:500 | 131kDa | Abcam, England |
| BC005321 | Anti-Arg-1 | Rabbit | WB 1:1000  IF and IHC 1:500 | 35kDa | ProteinTech,  China |
| 20700-1-AP | Anti-RunX2 | Rabbit | WB 1:1000  IF and IHC 1:500 | 57kDa | ProteinTech,  China |
| ab198228 | Anti- Osteocalcin | Rabbit | IHC 1:100 |  | Abcam, England |
| ab183597 | Anti-Apolipoprotein E | Rabbit | WB 1:1000  IF 1:500 | 36kDa | Abcam, England |
| ab315478 | Anti-MCP1(CCL2) | Rabbit | WB 1:1000  IF 1:500 | 11kDa | Abcam, England |
| ab109732 | Anti-FBP1 | Rabbit | WB 1:1000  IF 1:500 | 37kDa | Abcam, England |
| ab218237 | Anti-Osteopontin (SPP1) | Rabbit | WB 1:1000  IF 1:500 | 35kDa | Abcam, England |
