# Supplementary material for: Hydrogel-Wrapped Calendula officinalis L. extracellular vesicles - A novel approach to enhance fracture healing by Macrophage reprogramming
Source: Mater Today Bio. 2025 Nov 28;35:102592. doi: 10.1016/j.mtbio.2025.102592 (PMC12704079; doi:10.1016/j.mtbio.2025.102592)
Supplement: Multimedia component 2 [file mmc2.docx]

**Table S2.** Primer sequences of each gene.

| Target | Forward | Reverse |
| --- | --- | --- |
| SPP1 | AGCAGCTTTACAACAAATACCCAG | TACTTGGAAGGGTCTGTGGG |
| ACE | TCTGGCAGAACTTCACGGAC | TTGCTTAGCAGGGCGTTGTA |
| CCL2 | AGCAGCAAGTGTCCCAAAGA | GGTGTCTGGGGAAAGCTAGG |
| FN1 | AAGAAGGGCTCGTGTGACAG | TCTTGTCCTACATTCGGCGG |
| APOE | CCTTCCCCAGGAGCCGAC | GAATGTGACCAGCAACGCAG |
| MMP12 | GATGTGGAGTCCCCGATGTC | GGATTTGGCAAGCGTTGGTT |
| COL1A1 | TGACGAGACCAAGAACTGCC | GCACCATCATTTCCACGAGC |
| MMP14 | GGCGAGTATGCCACATACGA | GTACTCGCTATCCACTGCCC |
| FBP1 | CAGTCGGTCTGTCAGTCCTC | TGGGGCTCTTCTTGTTAGCG |
| SLCO2B1 | AGATCACCTGAGGCAGGGC | GCTCACGTTTTTCCTTGGGC |
| Arg-1 | CTCCAAGCCAAAGTCCTTAGAG | GGAGCTGTCATTAGGGACATCA |
| iNOS | GTTCTCAGCCCAACAATACAAGA | GTGGACGGGTCGATGTCAC |
| IL-10 | CTTACTGACTGGCATGAGGATCA | GCAGCTCTAGGAGCATGTGG |
| TNF-α | CGAGTGACAAGCCTGTAGCC | ACAAGGTACAACCCATCGGC |
| OCN | AAACATGGCAAGGTGTGTGA | AGGTGACCAGGACGTTTTTG |
| RunX2 | TCTTCCCAAAGCCAGAGCG | TGCCATTCGAGGTGGTCG |
| GAPDH | AATGGGCAGCCGTTAGGAAA | GCGCCCAATACGACCAAATC |
